# Expanding known viral diversity in plants: virome of 161 species alongside an ancient canal

**DOI:** 10.1186/s40793-022-00453-x

**Published:** 2022-11-27

**Authors:** Shixing Yang, Qingqing Mao, Yan Wang, Jingxian He, Jie Yang, Xu Chen, Yuqing Xiao, Yumin He, Min Zhao, Juan Lu, Zijun Yang, Ziyuan Dai, Qi Liu, Yuxin Yao, Xiang Lu, Hong Li, Rui Zhou, Jian Zeng, Wang Li, Chenglin Zhou, Xiaochun Wang, Quan Shen, Hui Xu, Xutao Deng, Eric Delwart, Tongling Shan, Wen Zhang

**Affiliations:** 1 Department of Laboratory Medicine, School of Medicine, Jiangsu University, Zhenjiang, 212013 Jiangsu China; 2 International Genome Center, Jiangsu University, Zhenjiang, 212013 Jiangsu China; 3 Suzhou Medical College of Soochow University, Suzhou, 215123 China; 4 Department of Laboratory Medicine, Jiangsu Taizhou People’s Hospital, Taizhou, 225300 Jiangsu China; 5 The Affiliated Hospital of Jiangsu University, Zhenjiang, 212001 Jiangsu China; 6 Vitalant Research Institute, San Francisco, CA 94118 USA; 7 Department of Laboratory Medicine, University of California, San Francisco, CA 94118 USA; 8 Shanghai Veterinary Research Institute, Chinese Academy of Agricultural Sciences, Shanghai, 200241 China

**Keywords:** Plant virome, Phytocommunity, Virus host switching, Co-infection, Phylogenetic analysis

## Abstract

**Background:**

Since viral metagenomic approach was applied to discover plant viruses for the first time in 2006, many plant viruses had been identified from cultivated and non-cultivated plants. These previous researches exposed that the viral communities (virome) of plants have still largely uncharacterized. Here, we investigated the virome in 161 species belonging to 38 plant orders found in a riverside ecosystem.

**Results:**

We identified 245 distinct plant-associated virus genomes (88 DNA and 157 RNA viruses) belonging to 27 known viral families, orders, or unclassified virus groups. Some viral genomes were sufficiently divergent to comprise new species, genera, families, or even orders. Some groups of viruses were detected that currently are only known to infect organisms other than plants. It indicates a wider host range for members of these clades than previously recognized theoretically. We cannot rule out that some viruses could be from plant contaminating organisms, although some methods were taken to get rid of them as much as possible. The same viral species could be found in different plants and co-infections were common.

**Conclusions:**

Our data describe a complex viral community within a single plant ecosystem and expand our understanding of plant-associated viral diversity and their possible host ranges.

**Supplementary Information:**

The online version contains supplementary material available at 10.1186/s40793-022-00453-x.

## Background

Much effort has been devoted to studying viruses associated with economically important or diseased plants which only comprise a minute fraction of all plant species, suggesting that a large gap exists in our overall understanding of viral diversity in uncultivated plants [1]. To gain a more objective view of plant virus diversity, it is therefore necessary to study viruses existing in wild plants. A viral metagenomics approach makes it possible to identify both already known as well as highly divergent viral genomes in wild plants. At present, four main classes of nucleic acids including total RNA or DNA, virion-associated nucleic acids (VANA) purified from virus-like particles, double-stranded RNAs (dsRNA), and virus-derived small interfering RNAs (siRNAs) are used for plant viral metagenomic approach [2, 3], they have their own advantages and disadvantages such as the total RNA/DNA approach is simplest, but it can obtain a very high proportion of non-virus sequences, viruses in thus samples may not be detected because of their low titers; although the process of the VANA approach is cumbersome, it can simultaneous detect both RNA and DNA viruses which are encapsulated; the dsRNA approach can give a very in-depth analysis of virus-specific sequences, but the main weakness of this approach is not suit for the detection of single negative strand RNA viruses and DNA viruses; the siRNA approach is very sensitive for detecting both known and unknown viruses within single plants, but this approach may miss persistent viruses and may be difficult to accurately assemble novel viruses. We combined and simplified the total RNA/DNA approach and the VANA approach for the virome study from clinical samples and animal tissue or fecal samples in our previous studies. A large number of virome data from animal and clinical samples were obtained using this method [4–9]. We found many plant virus sequences during the process of library data analysis from animal or human fecal samples, among which some plant virus sequence is low similarity with those known plant virus sequences. This provoked us to study plant viral virome of ecosystem using this approach.

The Zhenjiang ancient canal was built starting during the Qin Dynasty over 20 centuries ago and is 16 km long with an average width of 40 m. The Dingmao section of the canal is 2 km long and is flanked by wild plants, plus landscape plants and crops. This section of the canal was selected to collect plant leaves for studying plant virome and the relationship between plant viruses and the ecosystem. We collected leaf samples of all 161 plant species which is existing in this area. Using viral metagenomics, we investigated and compared the virus community in these wild and cultivated plants. This study will improve our understanding of plant virus diversity and help to identify the potential host of plant viruses theoretically.

## Materials and methods

### Plant leaf samples

The goal of this study was to investigate the virome of plant species in an ancient canal ecosystem in Zhenjiang City, Jiangsu Province, China. The Zhenjiang Ancient Canal has a history of more than 2000 years. It runs through the whole town of Zhenjiang from southeast to northwest and is 16 km long with an average width of 40 m. By investigating the riparian vegetation of the ancient canal, the Dingmao section of the canal was chosen to study as a representative section. It is 2 km long with lots of wild plants, some landscape plants, and crops on both riversides. In a total of 161 plant species belonging to 38 different orders, 6 classes (Coniferopsida, Cycadopsida, Dicotyledoneae, Filicopsida, Ginkgopsida, and Monocotyledoneae), and 3 phyla (Angiospermae, Gymnospermae, and Pteridophyta) were collected in this area for this study. The sampling sites for each plant species are labeled on the map with numbers corresponding to plant library numbers (Additional file 1: Table S1, and Additional file 2: Figure S1, Additional file 3: S2). Among those plant species, 72 are wild plants and 89 are cultivated plants including landscape plants and crops. During sampling, 3 leaves from three different individual plants belonging to the same species were respectively collected into disposable materials, before this step, distilled water (ddH2O) was used to clean the dust and other non-plant organisms on the leaf surface. Before viral metagenomic analysis, about 0.1 g leaf tissue sample of each plant was grounded using steel balls and re-suspended in 1 mL of phosphate-buffered saline (PBS) and vigorously vortexed for 5 min. The grounded samples were then frozen and thawed three times on dry ice. The supernatants were then collected after centrifugation (10 min, 15,000 × g) and stored at − 80℃ until use. Host species identification was initially identified using the APP “Picture This” which is an online plant encyclopedia and plant identifier, and further confirmed by experienced field biologists.

### Viral metagenomic analysis

About 300 μL supernatant from each of the three different plant samples in the same species was mixed into one sample pool and filtered through a 0.45-μm filter and centrifuged at 12,000 × g for 20 min at 4 °C to remove eukaryotic and bacterial cell-sized particles. Un-encapsidated nucleic acids were then digested by DNase and RNase at 37 °C for 60 min [10, 11]. Total nucleic acids were extracted as a mixed RNA/DNA solution using QiaAmp Mini Viral RNA kit (Qiagen) according to the manufacturer’s protocol. 161 libraries were constructed using Nextera XT DNA Sample Preparation Kit (Illumina). For bioinformatics analysis, paired-end reads of 250 bp generated by MiSeq were debarcoded using vendor software from Illumina. An in-house analysis pipeline running on a 32-node Linux cluster was used to process the data. Reads were considered duplicates if bases 5 to 55 were identical and only one random copy of duplicates was kept. Clonal reads were removed and low sequencing quality tails were trimmed using Phred quality score ten as the threshold. The unique read number of each library was shown in Additional file 1: Table S1. Adaptors were trimmed using the default parameters of VecScreen which is NCBI BLASTn with specialized parameters designed for adapter removal. The cleaned reads were de novo assembled within each barcode using the ENSEMBLE assembler [12]. Contigs and singlets reads are then matched against a customized viral proteome database using BLASTx with an E value cutoff of < 10 − 5, where the virus BLASTx database was compiled using NCBI virus reference proteome (ftp://ftp.ncbi.nih.gov/refseq/release/viral/) to which was added viral protein sequences from NCBI nr fasta file (based on annotation taxonomy in Virus Kingdom). Candidate viral hits are then compared to an in-house non-virus non-redundant (NVNR) protein database to remove false-positive viral hits, where the NVNR database was compiled using non-viral protein sequences extracted from NCBI nr fasta file (based on annotation taxonomy excluding Virus Kingdom). Contigs without significant BLASTx similarity to the viral proteome database are searched against viral protein families in vFam database [13] using HMMER3 with default parameters to detect remote viral protein similarities [14–16]. A web-based graphical user interface was developed to present users with the virus hits, along with taxonomy information and processing meta-information. The genome coverage of the target viruses were analyzed by Geneious v11.1.2 [17].

### Confirmation and extension of virus genomes

Viral contigs which might be from the same genome but without assembled overlaps were merged using the software Geneious v11.1.2 and primers bridge contigs were then designed. Gaps were filled by (RT-)PCR and Sanger sequencing. To confirm the assembly results of a full genome, reads were de novo assemble back to the full length genome using the low sensitivity/fastest parameter in Geneious 11.1.2. For genomes with novel structures, we verified the complete or near complete viral genome by designing overlapping primers based on the assembled sequences. For those viruses that firstly isolated from plants, we used PCR and Sanger sequencing to verify it’s accurate based on the assembled sequences.

### Confirmation of viral co-infection

In 7 libraries including pt065, pt067, pt110, pt111, pt112, pt119 and pt151, which have far more than three different viruses, showed evident co-infection in individual plant. To investigate the presence status of different viral strain in three individual plants from the same library, PCR and Sanger sequencing were performed on those from co-infected plants using specific primers designed based on the conserved domain sequences of these viruses.

### Phylogenetic analysis of viruses

Through analyzing the protein sequences obtained in this study, we divide them into three categories including RNA viruses, Parvovirus-like viruses, and CRESS DNA viruses. To infer the phylogenetic relationships, protein sequences of reference strains belonging to RNA viruses, Parvovirus-like viruses, and CRESS DNA viruses were downloaded from the NCBI GenBank database. For RNA viruses, the phylogenetic tree was constructed based on the RNA-dependent RNA polymerase (RdRp), for parvovirus-like viruses, the phylogenetic tree was constructed based on nonstructural protein (NS), for the CRESS DNA viruses, the phylogenetic tree was constructed based on the replication-associated protein (Rep) except for *Microviridae* viruses whose major capsid protein was used for the phylogenetic tree construction. The related protein sequences were firstly aligned using alignment program implemented in the CLC Genomics Workbench 10.0, the alignment result was further optimized using MUSCLE in MEGA v7.0 [18] and MAFFT v7.3.1 employing the E-INS-I alforithm [19]. Sites containing more than 50% gaps were temporarily removed from alignments. Bayesian inference trees were then constructed using MrBayes v3.2 [20]. The Markov chain was run for a maximum of 1 million generations, in which every 50 generations were sampled and the first 25% of Markov chain Monte Carlo (MCMC) samples were discarded as burn-in. The MrBayes runs were optimized by providing the ESS (all ESS value were > 100) of the MCMC. The amino acid substitution model used in the phylogenetic analysis is “prset aamodelpr = mixed”. The approximate family/genus of viruses obtained in this study was determined through the above tree, further constructed the detailed trees point at each virus family that are relatively closely related to the viruses discovered here using the same method. Maximum Likelihood trees were also constructed to confirm all the Bayesian inference trees using software Mega v7.0 or PhyML v3.0 [21].

### Virus genome annotation

Putative viral open reading frames (ORFs) were predicted by Geneious v11.1.2 with built-in parameters (Minimum size: 300; Genetic code: Standard; Start codons: ATG) [17], further were checked by comparing to related viruses by Blastp in NCBI. The annotations of these ORFs were based on comparisons to the Conserved Domain Database. Potential exon and intron of Genomovirus were predicted by Netgenes2 at http://www.cbs.dtu.dk/services/NetGene2/.

### Viral community analysis

The Alpha diversity analysis and the Species accumulated curve were used to reflect the richness and diversity of the communities using R v4.0.4 package vegan (v2.5–7, https://CRAN.R-project.org/package=vegan). Comparing differences between groups in viral communities was the analysis of Similarities (ANOSIM) and principal coordinate composition (PCoA) analysis under R v4.0.4 package vegan (v2.5–7, https://CRAN.R-project.org/package=vegan), permute (v2.5–7, https://CRAN.R-project.org/package=permute), and lattice (v2.5–7, https://CRAN.R-project.org/package=lattice). *P* values of these composition similarity analyses less than 0.05 were considered statistically significant. The viral community structure was visualized in a heatmap using R v4.0.4 package pheatmap (v1.0.12, https://CRAN.R-project.org/package=pheatmap). Linear discriminant analysis Effect Size (Lefse) which described and validated species that differ between two or more microbial communities was computed with an alpha value lower than 0.05 and an LDA score greater than 3.0 [22].

### Quality control in the nucleic acid manipulation

Standard precautions were used for all procedures to prevent cross-sample contamination and nucleic acid degradation. Mainly, aerosol filter pipet tips were used to reduce the possibility of sample cross-contamination, and all the materials (including microcentrifuge tubes, pipet tips, etc.) which directly contacted nucleic acid samples were RNase and DNase free. The nucleic acid samples were dissolved in DEPC treated water. In order to exclude the possibility of contamination with nucleic acids of parvovirus-like hybrid virus (PHV) and micirovirus present in the laboratory or from Qiagen nucleic acid extraction kits, the samples positive for the two types of viruses were chosen and the nucleic acids were re-extracted using Trizol reagent (Invitrogen). PCR using primers specific to those viruses confirmed their presence in the original biological samples. As a control, a library was also constructed using ddH_2_O as the sample which generated 13,228 raw reads and contained no viral reads based on BLASTx searching.

## Results and discussion

### Overall view of the virome

**Fig. 1:**
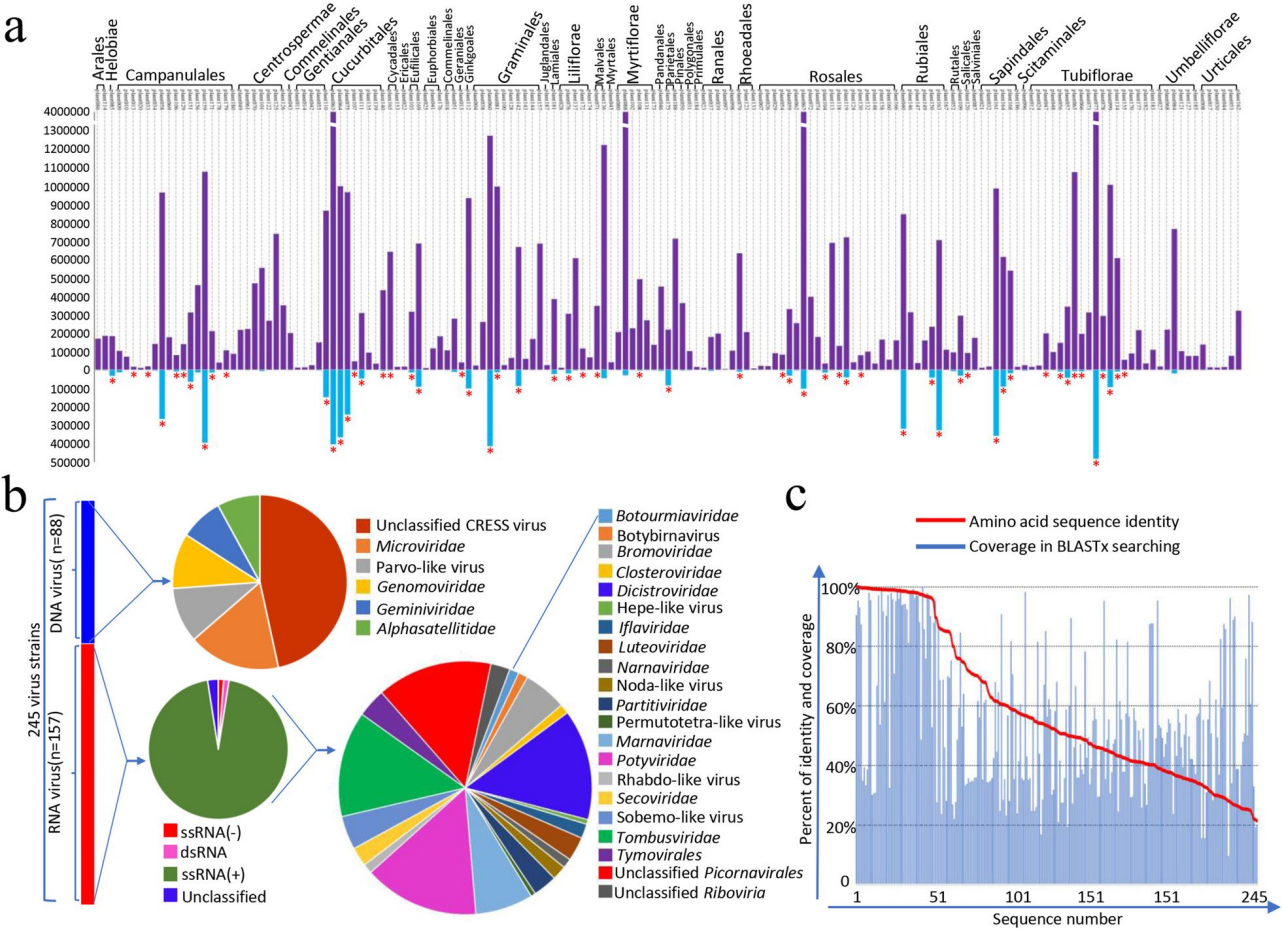
Identification of viruses in different species of plants. **a** The abundance of plant-associated viruses in different species of plant. The top graph shows the total number of unique reads in each library. The library IDs are shown on top of each bar, while the host Orders are shown above the bar graph. The bottom graph shows the number of virus hits passed NR filter in viral metagenomic bioinformatic analysis. The red asterisk shows those libraries from which we have determined complete or nearly complete genome of viruses. **b** The number and diversity of plant-associated viruses. The left histogram shows the numbers of DNA viruses (blue bar) and RNA viruses (red bar). The right pie charts show the virus classification identified in this study. **c** The amino acid sequence identity and coverage of plant-associated viruses with the best matched virus strains in BLASTx searching based on the 245 complete genome sequence determined in plant species

We performed a large-scale viral metagenomics survey of potential plant leaf-associated viruses in 161 plant species classified in 38 different orders, 6 classes (Coniferopsida, Cycadopsida, Dicotyledoneae, Filicopsida, Ginkgopsida, and Monocotyledoneae), and 4 phyla (Angiospermae, Gymnospermae, Pteridophyta, and Tracheophyta) including 89 wild plants and 72 wild types (Fig. 1a, Additional file 1: Table S1, and Additional file 2: Fig. S1). 161 viral metagenomics libraries were constructed (a single plant species per library). A total of 50,586,188 sequence reads were generated and among which 21,989,438 sequence reads had the best matches with viral proteins (E value cut-off < 10^5^). Of the 161 sets of sequenced, 147 contained sequences showing significant similarities to known viruses with viral reads consisting of 0.04% to 97.93% of the total reads. Fifty-two libraries contained > 50% of viral reads (Fig. 1a, Additional file 1: Table S1). A total of 245 viruses generated 202 complete genomes or 49 nearly complete genomes (sequence length > 70% of the genome), including 5 RNA viruses belonging to segmented viruses (Fig. 1b). From these data, 27 different groups of viruses were detected, including viruses belonging to 17 families, 1 genus (Botybirnavirus), and 9 unclassified groups including circular replication-associated protein encoding single-stranded DNA virus (CRESS DNA virus), Parvo-like virus, Hepe-like virus, Noda-like virus, Permutotetra-like virus, Rhabdo-like virus, Sobemo-like virus, unclassified members of Picornavirales order*,* and unclassified members of the Riboviria realm (potential new orders) (Fig. 1b, and Additional file 1: Table S1). BLASTx search with these 245 viruses revealed that 61 shared < 40% amino acid sequence identities with their best matches in GenBank (Fig. 1c, and Additional file 4: Table S2), suggesting that these genomes could be considered as members of novel virus genera or families.

### Differences in viral communities between cultivated and wild plants

**Fig. 2:**
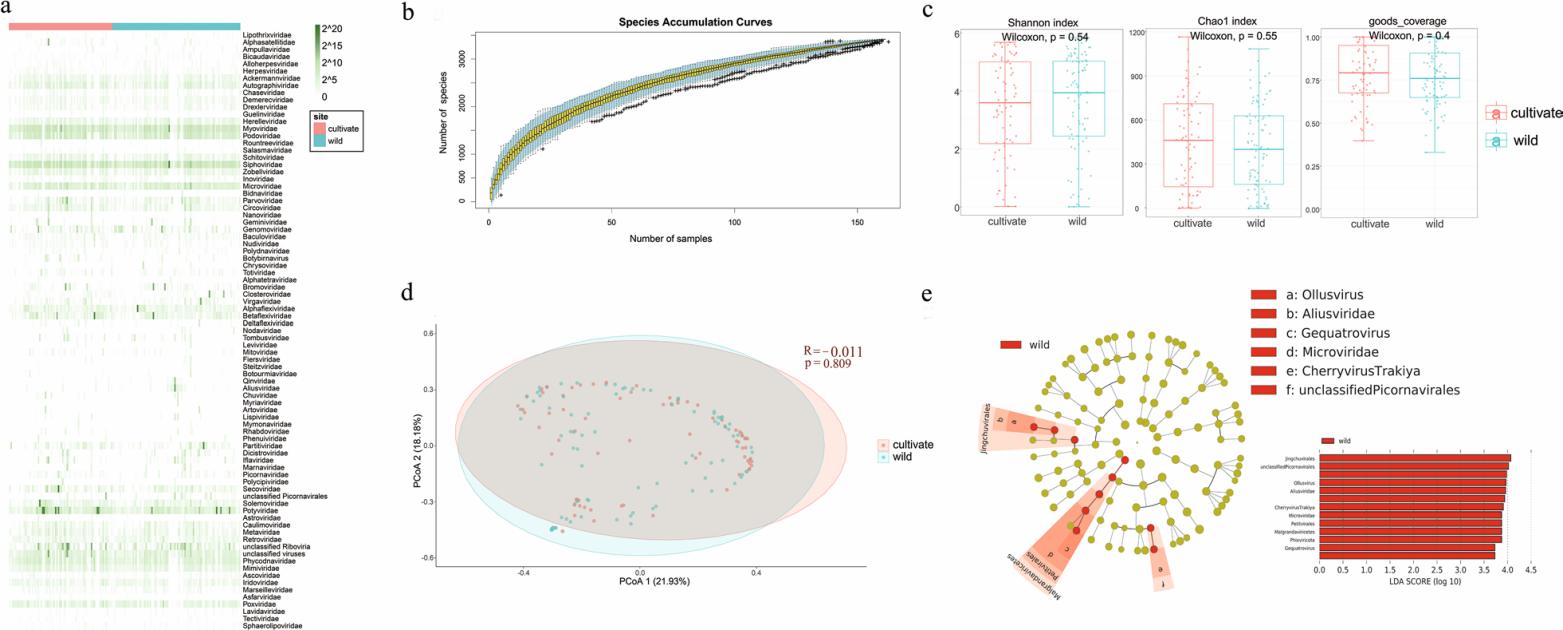
Analysis of virus community diversity in cultivated and wild plants. **a** Taxonomic analyses at the family level. The heatmap shows the reads counts of each virus family on a log2 scale. Plant types are indicated by the corresponding colors (see color legend). The row name on the right represents the name of the virus family. **b** Species accumulation curve. The abscissa represents the number of libraries, and the ordinate represents the number of species found. The blue shading indicates the 95% confidence interval. **c** Comparison of alpha diversity between the two groups (Shannon index, Chao1 index, and goods_coverage). The horizontal bars inside boxes represent medians. The tops and bottoms of boxes represent the 75th and 25th percentiles, respectively. The upper and lower whiskers extend to data no more than 1.5 × the interquartile range from the upper edge and lower edge of the box, respectively. The plant types are indicated with the corresponding colors (see color legend). **d** Principal coordinates (PCoA) analysis. The PCoA analysis shows the differences in species composition based on the Bray–Curtis ecological distance matrix. The *P*-value is calculated by ANOSIM. **e** Linear discriminant analysis Effect Size (Lefse). Circles radiating from inside to outside represent taxonomic classes from phylum to genus. Each small circle at a different taxonomic level represents a taxon at that level, and the small circle diameter size is proportional to the relative abundance size. Species without significant difference are uniformly colored yellow, and differential species Biomarker follows the group for staining. Red nodes indicate microbial groups that play an important role in red groups. Only taxa with LDA values of 3.0 or higher are shown

The heatmap revealed that the distribution of viral reads in the two groups of plants showed little difference at the level of the viral family and the dominant viral families were basically the same (Fig. 2a). The species accumulation curve gradually showed placid with the increase of the number of sampling (Fig. 2b). It demonstrated that the sample volume in this study was large enough to capture almost all viral species. To further confirm if there are viral composition differences between two different groups, we undergone the Alpha diversity analysis (including Shannon index, Chao1 index, and goods_coverage index), and Beta diversity analysis (including Principal co-ordinates analysis (PCoA)). Those Alpha diversity analysis indicated that the viral compositions of cultivated and wild plants had no significant difference (*P* > 0.05) (Fig. 2c). The PCoA analysis gave the similar result (Fig. 2d), in which the ellipse representing the wild plants practically overlapped the other ellipse representing the cultivated plants (*P* > 0.05). Further, we performed the Linear discriminant analysis Effect Size (LEfSe) to find the species with significant difference in abundance (i.e. biomaker) in different groups. The LEfSe analysis (Fig. 2e) showed that compared with cultivated plants, there were differences in the three virus orders of wild plants (including *Jingchuviruales*, *Petitvirales*, and unclassified *Picornavirales*). To sum up, the virus composition of cultivated and wild plants beside Zhenjiang ancient canal was only a little different and almost negligible. Since viral metagenomics was first used to study plant viruses in 2006, a larger number of plant viruses have been identified from cultivated plants [23–27]. Cultivated crop species became the main source of virus infection, just as plant viromes revealed in The Nature Conservancy's Tallgrass Prairie Preserve of Oklahoma by Melcher and co-workers, where the 77% is available virus information derived from cultivated crop species, while only 6% of virus information is obtained from wild plants [23]. Here, our result showed no significant difference between the two groups (Fig. 2, and Additional file 1: Table S1). And there were only minor differences in the composition of the viral community between the two groups of plants. The virus proportion difference in the two studies might be due to the differences in sampling sites or samples treatment method where the former only used dsRNA for library construction, while this study extracted the total RNA/DNA after enriching viral particles through filtration and enzyme digestion. Our data suggested that cultivation mode had no discernable effect on the plants’ susceptibility to virus infection.

### Plants as hosts of diverse viruses

In this study, we detected and characterized 27 different groups of viruses including many viruses not previously reported from plants (temporarily named plant-associated viruses) and confirmed plant viruses (Figs. 3, 4 and 5, see Additional file 3: Figs. S3-S31 for detailed phylogenies).

**Fig. 3:**
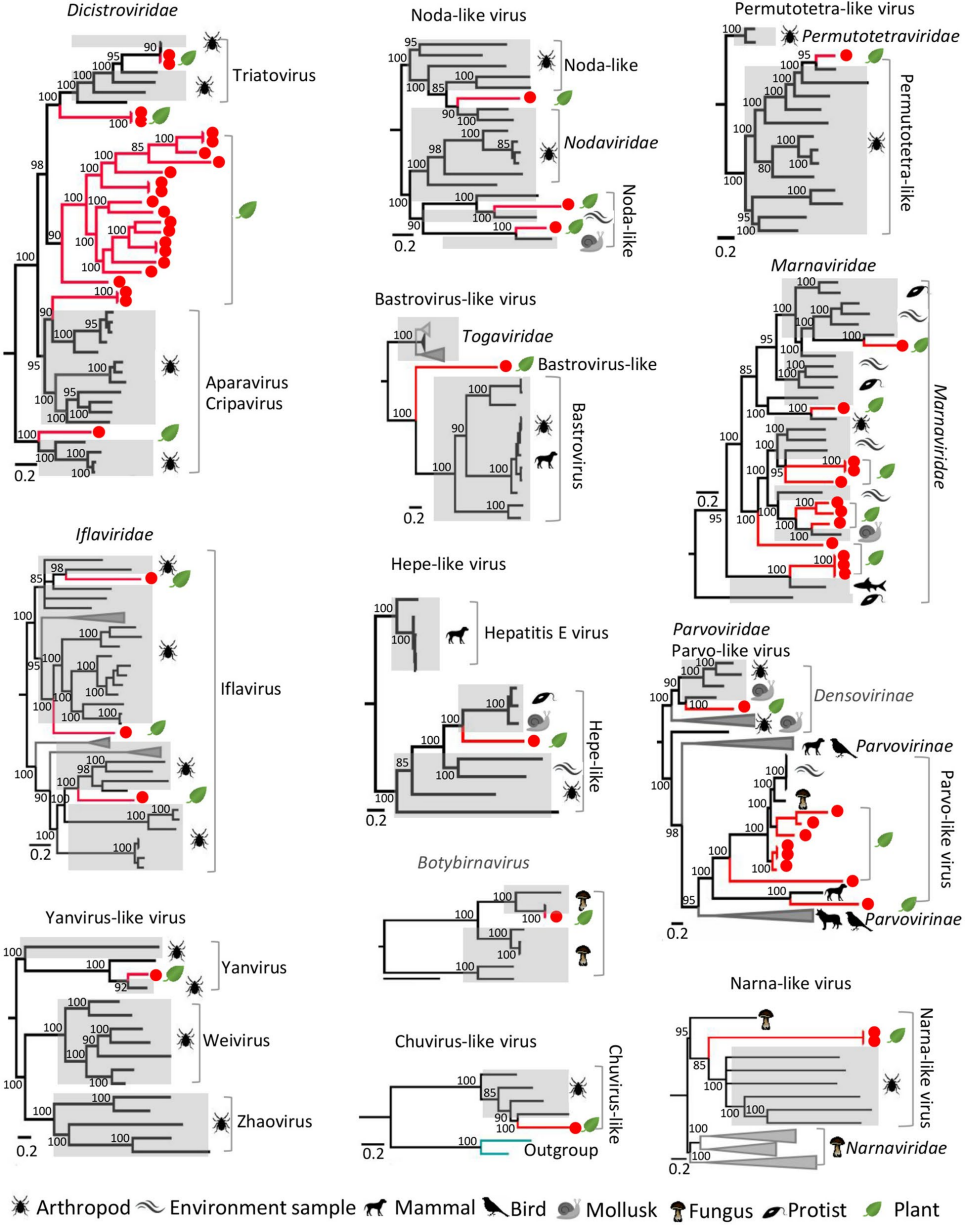
Phylogenies of viral genomes identified from plants. Twelve Bayesian inference trees were constructed using MrBayes v3.2 based on virus RdRp domain of RNA viruses or NS protein of parvovirus-like viruses, within each tree, the viruses found in this study are marked with red line. Hosts are indicated with different silhouette of mammal, bird, arthropod, plant leaf, or waves standing for virus environmental source. The name of the virus family or genus is shown on the right side of each cluster. Each scale bar indicates 0.5 amino acid substitutions per site. The posterior probability scores is labelled on each nodes of the phylogenetic tree as the percentage value

**Fig. 4:**
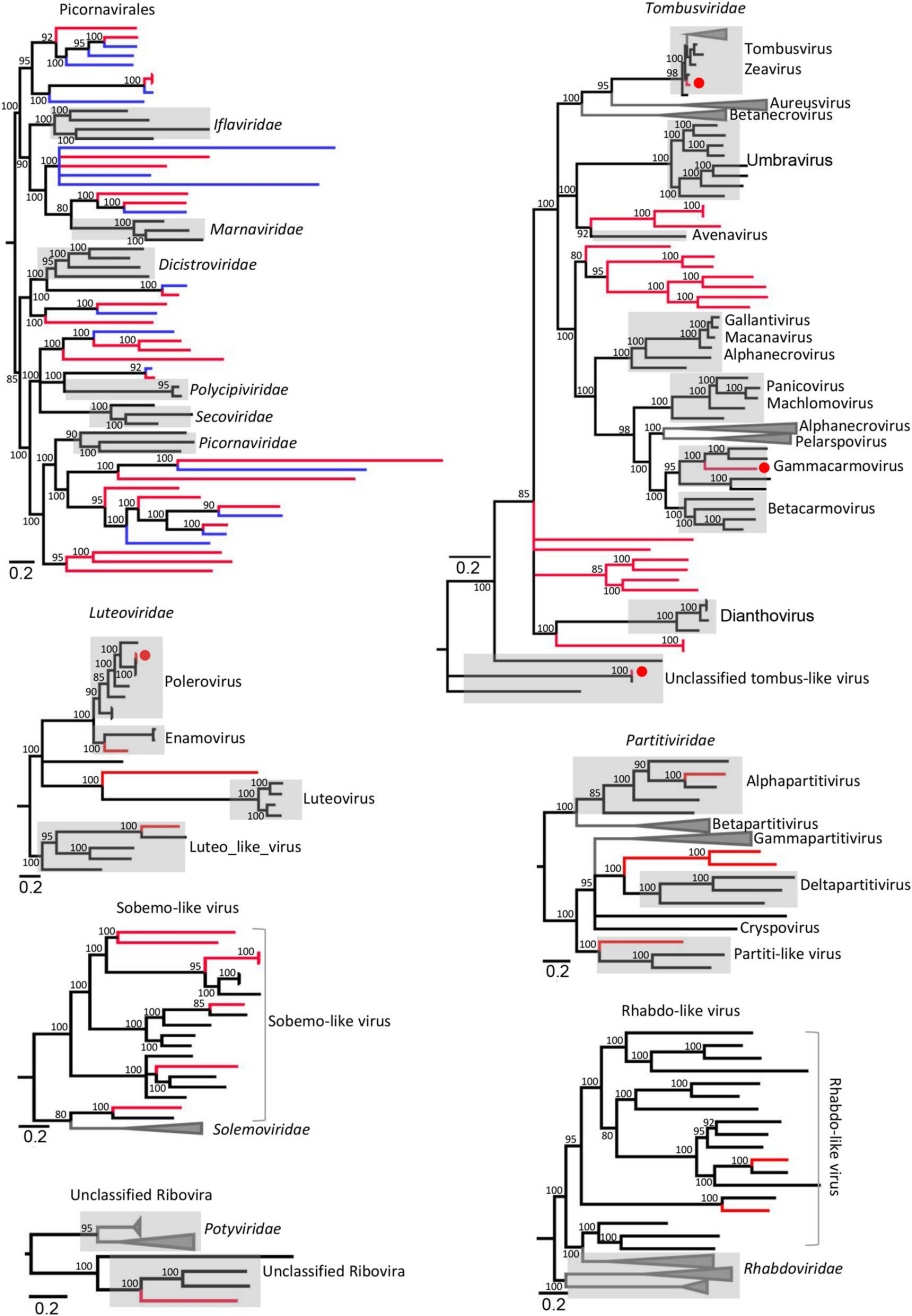
The phylogenies of potentially new viruses. Seven Bayesian inference trees were constructed using MrBayes v3.2 based on virus RdRp domains, within each tree, the viruses found in this study are marked with red line. In the phylogenetic tree of Picornavirales the best matched virus based BLASTp searching using RdRp sequence of each novel virus are labeled with blue color. The name of the virus family or genus is shown on the right side of each cluster. Each scale bar indicates 0.2 amino acid substitutions per site. The posterior probability scores is labelled on each nodes of the phylogenetic tree as the percentage value

Those plant-associated viruses fall into different virus families whose members hosted by invertebrate, vertebrate, insect, bacteria, and fungus. Members of the order *Picornavirales* including dicistroviruses, iflaviruses, marnaviruses were detected here and accounted for a large proportion. The family *Dicistroviridae* is a group of viruses currently composed of 3 genera, whose natural hosts are invertebrates, including aphids, leafhoppers, flies, bees, ants, and silkworms [28]. Here, we assembled 23 genomes from 10 different species of plants. 7 viruses were grouped into three previously classified genera, while the other 16 viruses were clustered into a separate group genetically far from the three known genera (Fig. 3, Additional file 1: Table S1, see Additional file 3: Fig. S3). Although these 16 viruses shared < 50% RdRp protein sequence similarities to their best BLASTp matches in GenBank, they showed typical genome organizations of dicistroviruses (Additional file 3: Fig. S4). It suggested that these 16 dicistroviruses might belong to a putative new genus of the family *Dicistroviridae*. The family *Iflaviridae* is a member of the order *Picornavirales*, which have also all been isolated from arthropods. In this study, three divergent iflavirus genomes were obtained from three different species of plants, all of which clustered within genus *Iflavirus* in phylogenetic tree (Fig. 3, and Additional file 3: Fig. S5). Previous studies have shown that in arthropods, infection acquisition and transmission of dicistrovirus or iflavirus are prominently accomplished by ingestion and spread from the alimentary canal [29]. In addition, vertical and sexual transmission has been reported among invertebrates for some iflaviruses [30, 31]. Based on the transmission pattern of dicistrovirus and iflavirus in arthropod, the identification of dicistroviruses in plants may be come from virus-contaminated feces of insects shed onto the plant leaf surface. *Marnaviridae* is a newly defined virus family in order *Picornavirales*, the currently characterized representative member being *Heterosigma akashiwo* RNA virus, isolated from *Heterosigma akashiwo* algae in ocean water [32]. Closely related viruses have been identified in ocean marine environments [33]. Here, we identified 12 marnaviruses from 5 different species of plants that shared 30–60% sequence identities based on pairwise comparison of polyprotein and showed typical genome organization of *Marnaviridae* (Fig. 3, and Additional file 3: Fig. S6). Phylogenetic analysis showed that the 12 marnaviruses grouped well into the cluster of genus *Marnavirus* within *Marnaviridae* (Fig. 3, and Additional file 3: Fig. S7). Because all 12 marnaviruses isolated from 5 different terrestrial plants are not aquatic plants and algae in this study, we inferred that plants were capable of hosting some members in the family *Marnaviridae*. In addition, another 24 divergent picorna-like viruses were genetically distinct from the defined families in the order *Picornavirales* (Fig. 4, and Additional file 3: Fig. S8). Besides viruses belonging to the order *Picornavirales*, another 4 groups of divergent viruses including Noda-like virus, Permutotetra-like virus, Yanvirus-like virus, and Chuvirus-like virus were also detected here (Fig. 3, and Additional file 3: Figs. S9-S12). These viruses were recently reported from invertebrates' meta-transcriptomes, and vertebrates and environment samples [34–36]. Since some vertebrates and invertebrates feed on plants, it is reasonable to speculate that these viruses previously discovered may have originated from plants.

To our surprise, three groups of viruses mainly hosted by vertebrates were detected here. One group were parvovirus-like viruses including 7 virus strains similarity to parvovirus-like hybrid virus (PHV) and 2 viruses showing close relationships to densovirus (Fig. 3, and Additional file 3: Fig. S13). These plant PHV genomes encoded two major forward-direction ORFs encoding the replication and capsid proteins (Additional file 3: Fig. S14), which is characteristic of viruses in the family *Parvoviridae*. The 7 PHVs detected in plants were grouped in two different clusters, sharing sequence similarities of 50%-67% to other PHVs based on the replication protein sequences. PHV is a type of highly divergent DNA virus that was recently discovered and phylogenetically located at the interface between the *Parvoviridae* and *Circoviridae* [37, 38]. PHV was first detected in Chinese patients with seronegative (non-A-E) hepatitis [37], the exact origin of the virus was eventually traced to contaminated silica-binding spin columns used for nucleic acid extraction by Charles and co-workers. Through analysis of environmental metagenome libraries detected PHV sequences in coastal marine waters of North America, they suggested that a potential association between PHV and diatoms (algae) that generate the silica matrix used in the spin columns may have resulted in inadvertent viral contamination during manufacture. But PHVs in our study shared sequence similarities of 50–67% to other PHVs, and were only detected from 5 libraries. We were not sure whether these viruses came from contaminated experimental materials. The other were bastroviruses which were previously reported in feces of mammals (including human) and shows a distant relationship to astroviruses [39, 40]. A species of plant (*Solanum melongena*) was positive for virus genome sequence showing 25% RdRp sequence similarity to that of bastrovirus in this study (Fig. 3, and Additional file 3: Fig. S15). Detecting this highly divergent bastrovirus-like virus in plants may imply bastrovirus has wide hosts such as vertebrates, invertebrates, and plants also be not excluded. Another species of plant was positive for hepe-like virus, which has been reported in mammals, invertebrates, protists, and different environments [34, 36, 41, 42]. This hepe-like virus from plants grouped with other hepe-like viruses from different types of organism and environment samples and shared similar genome organization (Fig. 3, and Additional file 3: Fig. S16).

Two types of viruses, botybirnavirus and narna-like virus, which were considered to be viruses of fungi [43, 44] and more recently *Caenorhabditis* nematodes [45], were detected in two species of plants, respectively (Fig. 3, and Additional file 3: Figs. S17–S18). The botybirnavirus showed high sequence identity (96.4%) to fungi batybirnavirus based on RdRp protein sequence. The two narna-like viruses from 2 different species of plants shared 99.9% nucleotide sequence identity based on RdRp protein sequences and were divergent from previous narna-like viruses. In addition, fifteen genomes showing sequence similarity to viruses in the family *Microviridae* were detected in three different species of plants (Additional file 3: Fig. S19). Many studies have demonstrated the ubiquity of *Microviridae* genomes across habitats (marine, freshwater, wastewater, sediment) and global regions (Antarctic to subtropical), especially those related to the *Gokushovirinae* lineage [46–49], which infect obligate intracellular parasites, members of the bacterial genera Chlamydia, Bdellovibrio and Spiroplasma [50]. The closest non-plant-infecting relatives of some genomes from plants reported here tended to infect arthropods, fungi and bacterial. Currently, plant-associated viruses may therefore have originated from viruses that once infected non-plant organisms (or vice versa). Further, the hypothesis that some plant viruses may have originated from arthropod viruses is also plausible as some viruses infecting arthropods can also infect plants. For example, flock house virus (in the *Nodaviridae* family) infects arthropods but can also systemically infect plants when it is complemented with the movement proteins of either tobacco mosaic virus or red clover necrotic mosaic virus (both of which are plant viruses) [51]. For better host assignment of these plant-associated viruses which cannot be confirmed as plant-infecting ones (e.g. Marnavirus, Picorna-like virus, unclassified CRESS-DNA virus, Batro-like virus, Hepe-like virus, et al.), we compared (by using BLASTx searching) the contigs assembled from the NGS data of each plant species (those containing possible non-plant-infecting virus) against the total mitochondrial proteome database that were downloaded from GenBank. Here, we used mitochondrial proteome and performed BLASTx search, because mitochondrial gene is a good hallmark for species classification, and the library may contain novel non-plant species. The searching results were showed in a heatmap (Additional file 5: Fig. S32), which indicated that although some libraries contain a small number of contigs showing significant sequence similarity to non-plant species such as algae, protozoan, fungi, and insect, most sequence reads were from plant species.

Many types of typical plant viruses belonging to the *Potyviridae*, *Bromoviridae*, *Closteroviridae*, *Comoviridae*, and *Botourmiaviridae* families and *Tymovirales* order were also detected in several species of plants. These plant viruses were genetically close to previously described viruses (Additional file 3: Figs. S20–S25), indicating that typical plant virus infections were readily detected in this plant ecosystem. In addition, plant viruses belonging to the *Tombusviridae*, *Luteoviridae*, *Partitiviridae*, *Solemoviridae*, and *Rhabdoviridae* families and unclassified *Riboviria* realm were detected in different plants in present study. Some of these viruses were highly divergent and could not be classified into defined genera, which may belong to new genus in different virus families (Fig. 4 and Additional file 3: Figs. S26–S31). The enveloped viruses were rarely detected in plants in previous studies. Here, we obtained two Rhabdo-like virus genomes which belonged to enveloped viruses from two different plants. The proportion of the enveloped viruses was about 0.82% (2/245) of total viruses. Because some members of the family *Rhabdoviridae* hosted in plants, novel Rhabdo-like viruses identified here may be hosted in plants. Although most plant viruses are not pathogens, some plant viruses can infect and cause plant disease. Emerging diseases have garnered the most attention because of damage to economically important food and ornamental plant species. Important examples of viruses that are responsible for well-studied emerging diseases include cassava-infecting begomoviruses (in the *Geminiviridae* family) [52], closteroviruses causing grapevine leafroll disease [53], luteoviruses such as barley yellow dwarf virus [54] and sobemoviruses such as rice yellow mottle virus [55]. Relatives of all these pathogenic viruses were detected in this study in apparently healthy plants from diverse genera or families. The relatively unbiased sequencing of viral genomes within entire environments as performed here is changing the perspective of viruses from agents of disease to common components of ecosystems, as the plant tissue samples studied were all from apparently healthy plants.

### Plant CRESS virus

**Fig. 5:**
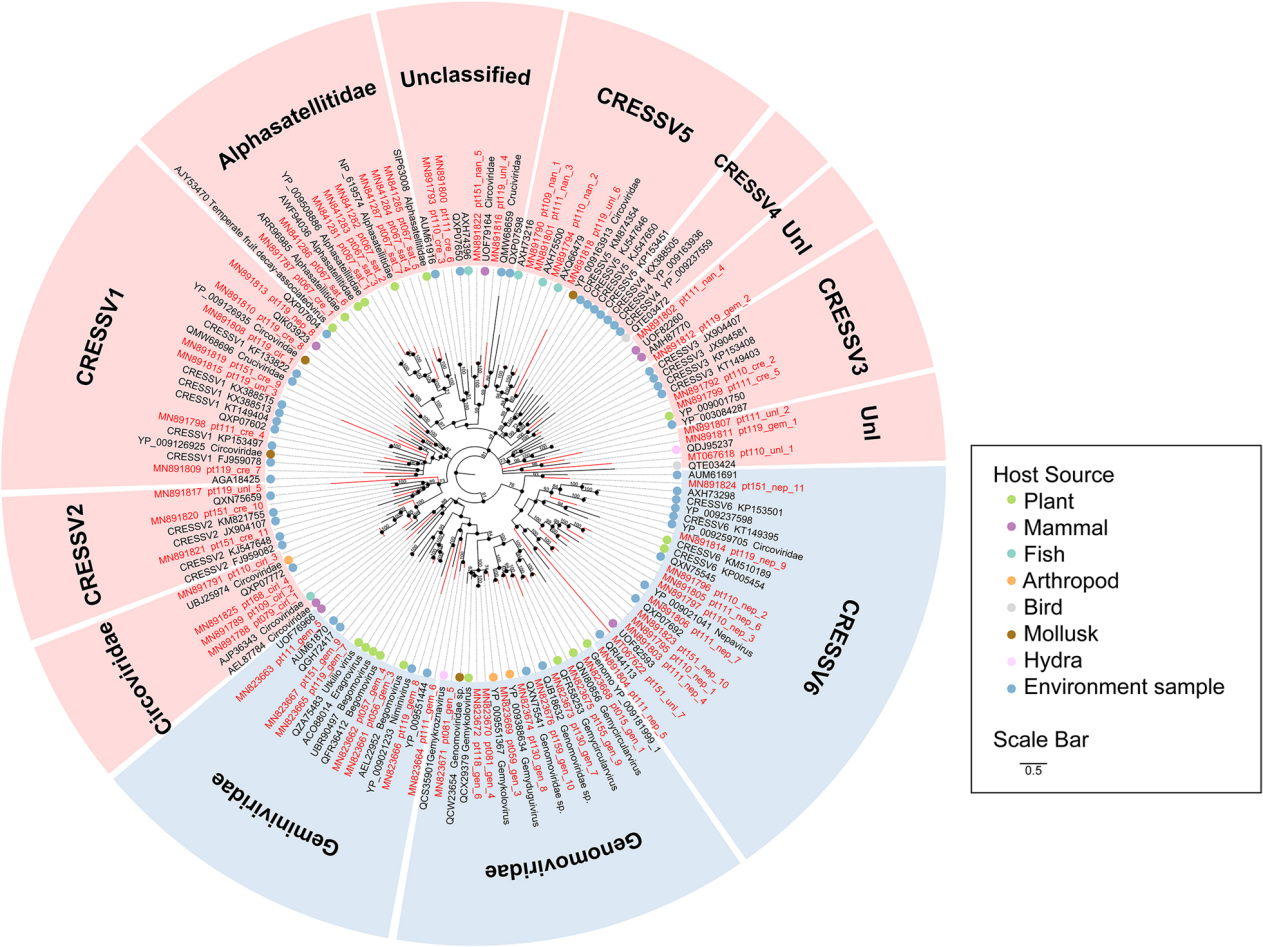
The phylogenetic tree of potentially new CRESS DNA viruses. The phylogenetic tree was established using MrBayes v3.2 based on Rep amino acid sequences, and the viruses found in this study are indicated by red lines. The host source of reference sequences are marked with corresponding colors (see color legend). Different virus groups are labeled on the diagram. The size of the black dots on nodes is positively correlated with the corresponding bootstrap score. The scale bar indicates 0.5 amino acid substitutions per site. The posterior probability scores is labelled on each nodes of the phylogenetic tree as the percentage value

CRESS DNA virus is the informal name of several groups of single-stranded (ss) DNA viruses that have circular and replication-associated protein (Rep) encoding genome, which show high diversity and abundance in various habitats [56, 57]. Although there are currently several established CRESS DNA virus families including *Bacilladnaviridae*, *Circoviridae*, *Geminiviridae*, *Genomoviridae*, *Redondoviridae*, *Nanoviridae*, and *Smacoviridae*, a large number of novel CRESS DNA viruses have been discovered recently and have not been formally classified, for which the hosts are currently unknown [57–59]. Among these well-defined CRESS DNA virus families, *Geminiviridae* and *Nanoviridae* are two plant-infecting members, which also help the replication and package of a satellite virus: *Alphasatellitidae,* another type of circular ssDNA genome [60]. Members of the *Circoviridae* such as porcine circoviruses infect vertebrates. Here, we determined 64 circular genomes from plant leaves, among which 7 were genetically close to *Geminiviridae*, 9 grouped well into the family *Genomoviridae*, 7 clustered closely to known sequences of *Alphasatellitidae*, with the remaining 41 showing significant sequence similarity to unclassified CRESS DNA viruses (Fig. 5). Most of the CRESS DNA viruses characterized in the present study best matched unclassified CRESS DNA genomes from environmental samples, mammalian feces, and arthropods, it is conceivable that some of these unclassified CRESS DNA viruses infect plants and were contaminants in feces or the gut content of arthropods. In previous studies, some researchers found that contaminating CRESS DNA virus sequences originated from reagent contamination. To confirmed the existence of CRESS-DNA viruses in the plant samples, we randomly selected 10 CRESS DNA viruses and designed PCR primers and conducted PCR to test whether these viruses genes were present in the samples, where the PCR templates were nucleic acid re-extracted from the untreated original sample pools using Trizol regents. The PCR products were T-A cloned and Sanger sequenced. Results confirmed the existence of these virus genomes in the original samples. Since we didn’t detect all CRESS DNA viruses using PCR method, we could not rule out whether the remaining CRESS DNA viruses came from contaminated reagents. In any case, reagent contamination should be given enough attention for virus metagenomics study.

### Co-infection of plant viruses

**Fig. 6:**
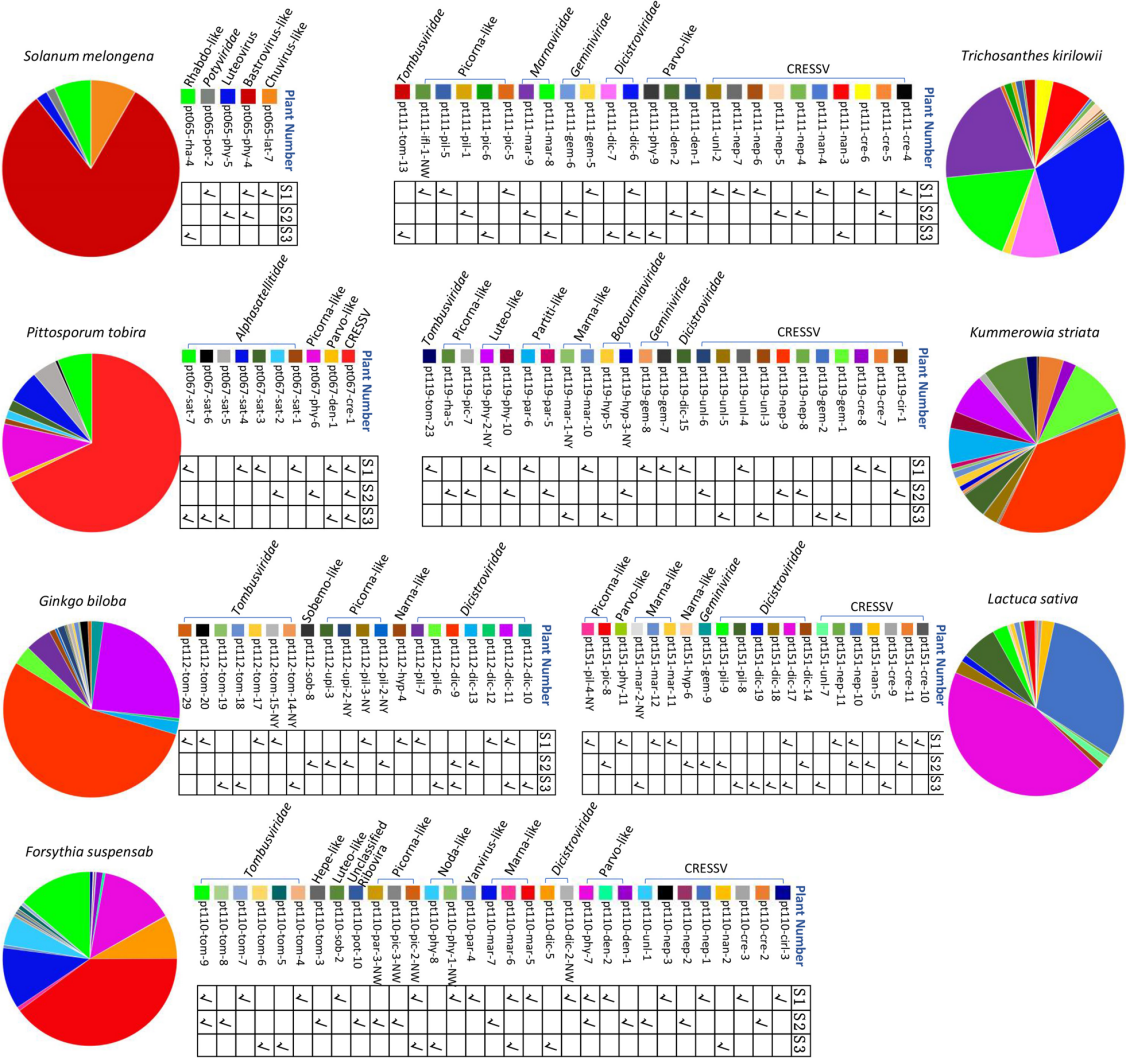
Co-infecting viruses in plants. Pie charts describe the viruses with complete genome in those libraries containing more than 3 different viruses. Sector area in each pie chart represents the proportion of the number of reads mapped to the complete viral genome in the library. Three individual plant in the same plant species are marked with S1, S2 and S3, respectively. Check marks below virus names show positive of virus in (RT-)PCR screening

Co-infection of hosts by two or more plant viruses is common in both agricultural crops [61, 62] and natural plant communities [63]. In the present study, co-infection of plant viruses was commonly observed, where 73 out of 161 (45.3%) libraries contained > 3 different virus types (or families) (Additional file 1: Table S1), suggesting co-infection of viruses existed in nearly half of the plants in this ecosystem as each library consisted of samples from three different individual plant. Considering the same virus families or type in a single library may contain different virus strain or type, the rate of co-infection is likely to be higher than 45.3%. Among the 245 genomes we determined from these plants, some genomes were from the same libraries which allow us to investigate the co-infection of certain viruses in specific species of plants. As shown in Fig. 6, PCR screening of different virus genomes in 7 different species of plants revealed that most of (20/21) the individual plant contained > 2 different types of viruses, where one plant species of *Forsythia suspensab* even carried 16 viruses belonging to 12 different families. The wide presence of apparent viral co-infections in these plants in a single ecosystem may lead to interactions between viruses that could influence disease development in individual plants.

## Conclusion

Our study showed that a number of genomes from viral families not known to infect plants can be found in plants, in addition to those confirmed plant viruses. Furthermore, several genetically close or identical viruses were detected in plants from different species, suggesting cross-plant species transmission or multiple hosts of the same virus. No significant difference in virus composition between cultured plant group and wild plant group in the same ecosystem in this study reflected the influence of ecosystem on virus composition of local plants. Because plant viruses can be transmitted through various media including arthropod, insect, water or even wind, we cannot determine whether the plant viruses detected here came from remote areas or only within this plant locations. Further study with larger sample size and sampling area should be performed to monitor more relevant plant viruses, so as to better understand the accurate host of theses novel plant-associated viruses. This study expands our understanding of plant-associated viral diversity, provides useful information for monitoring the health of these plants, and may aid in the prevention and treatment of viral diseases in local plants.

## Supplementary Information

**Additional file 1.** **Table S1**: Plant species and library information.

**Additional file 2. Fig. S1**: Pictures of plant strains or leaves collected in the present study.

**Additional file 3. Fig. S2-31**: The map for sampling sit of plant samples in this study and the detailed phylogenetic tree and genome structure of different viruses in this study.

**Additional file 4. Table S2**: Information of viruses with complete genomes determined in the present study.

**Additional file 5. Fig. S32**: The host assignment of plant-associated viruses by using BLASTx searching the contigs assembled from the NGS data of each plant species (those containing possible non-plant-infecting virus) against the total mitochondrial proteome database that were downloaded from GenBank.

## Data Availability

All data supporting the findings of this study are available within the paper and its Supplementary Files. All complete or partial viral genome obtained in this study were deposited in GenBank with the accession numbers MN722411-MN722420, MN723593-MN723599, MN729612-MN729623, MN728806-MN728814, MN724250-MN724258, MN814305-MN814321, MN831436-MN831448, MN823661-MN823692, MN841281-MN841303, MN832441-MN832474, MN862333-MN862357, MN891787-MN891825, MT067617-MT067623 and MT134328 (See detailed information in Additional file 4: Table S2). The raw sequence reads generated here were deposited into the Sequence Read Archive of GenBank database and the accession nos. are shown Additional file 1: Table S1.
