# Supplementary material for: Expanding known viral diversity in plants: virome of 161 species alongside an ancient canal
Source: Environ Microbiome. 2022 Nov 27;17:58. doi: 10.1186/s40793-022-00453-x (PMC9703751; doi:10.1186/s40793-022-00453-x)
Supplement: Supplementary file 2 — Additional file 2. Fig. S1: Pictures of plant strains or leaves collected in the present study. [file 40793_2022_453_MOESM2_ESM.pdf]

Supplementary Figure 1.Pictures of plant strains or leaves collected in the present study

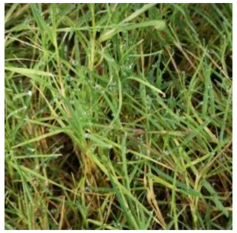	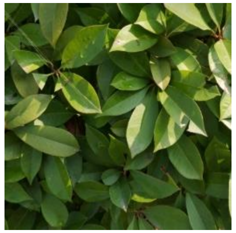	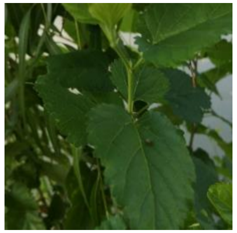	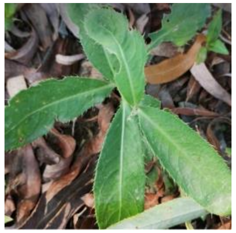	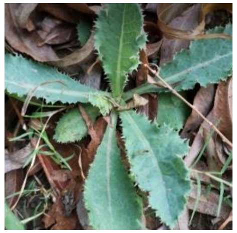	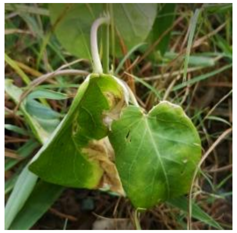	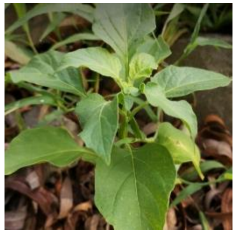
plant004, Cynodon dactylon (L.) Pers.	plant007, Photinia serrulata Lindl.	plant008, Morus alba L.	plant009, Cirsium setosum (Willd.) MB.	plant010, Cirsium japonicum Fisch. ex DC.	plant011, Metaplexis japonica (Thunb.) Makino	plant012, Solanum nigrum L.
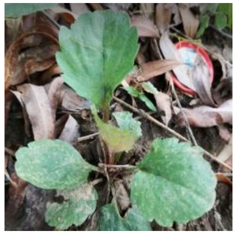	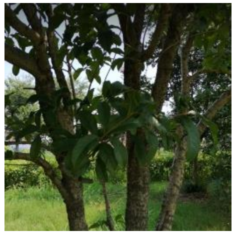	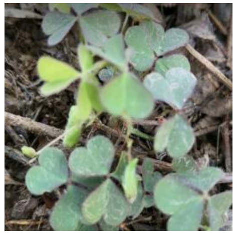	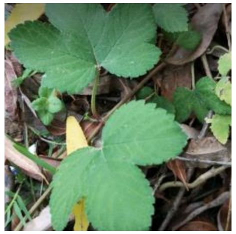	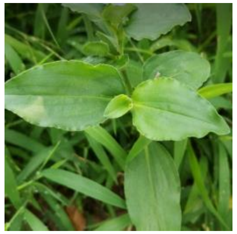	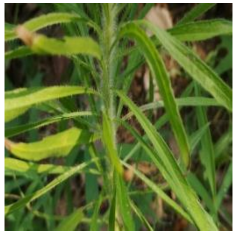	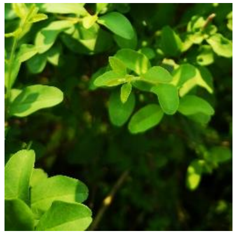
plant013, Erigeron annuus (L.) Pers.	plant014, Osmanthus fragrans (Thunb.) Lour.	plant015, Oxalis corniculata L.	plant017, Humulus scandens	plant018, Commelina communis	plant020, Conyza canadensis (L.) Cronq.	plant021, Buxus sinica (Rehd. et Wils.) Cheng
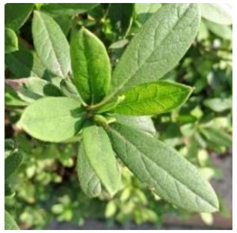	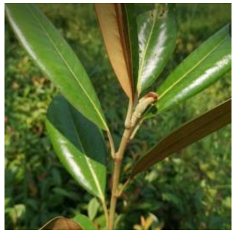	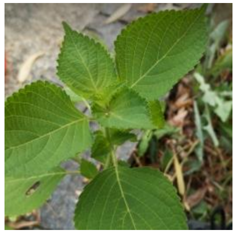	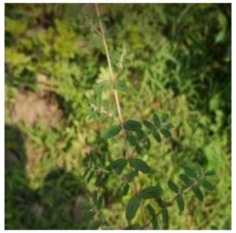	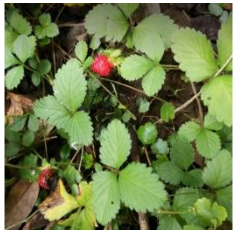	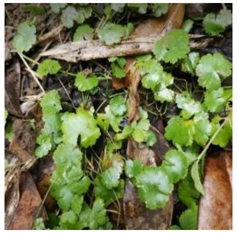	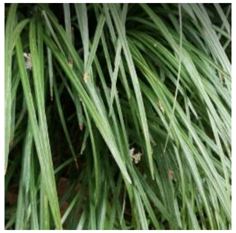
plant022, Rhododendron simsii Planch.	plant023, Magnolia grandiflora L.	plant024, Perilla frutescens (L.) Britt.	plant025, Humifuse Euphorbia Willd. ex	plant026, Duchesnea indica (Andr.) Focke	plant027, Hydrocotyle sibthorpioides Lam.	plant028, Ophiopogon bodinieri

			Schlecht.			
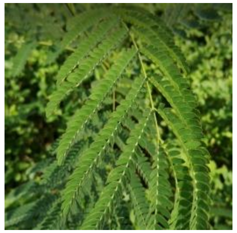	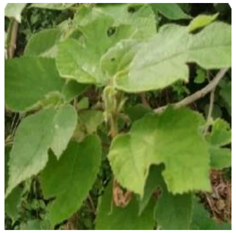	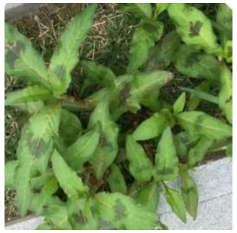	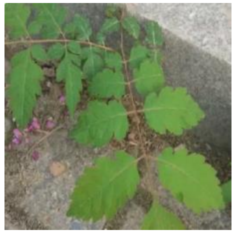	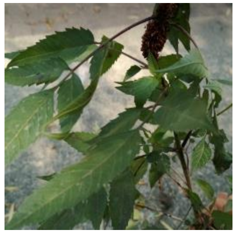	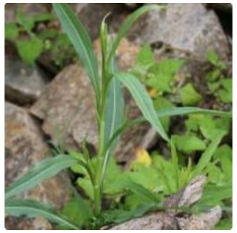	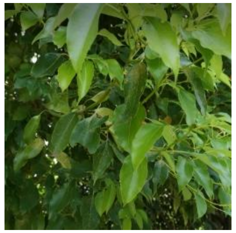
plant029, Albizia julibrissin Durazz.	plant030, Broussonetia papyrifera (Linn.) ex Vent.	plant031, Polygonum persicaria L.	plant032, Koelreuteria paniculata Laxm.	plant033, Bidens pilosa L.	plant034, Aster subulatus Michx.	plant037, Cinnamomum camphora (L.) presl
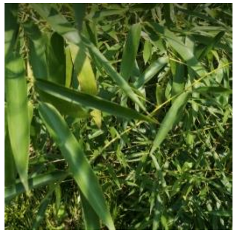	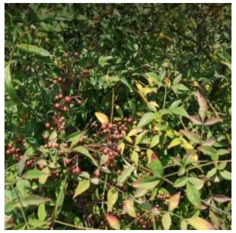	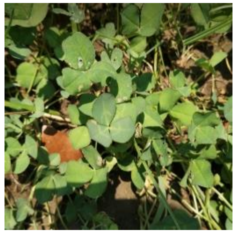	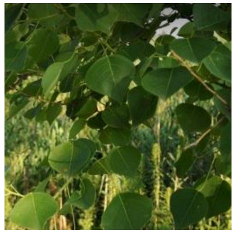	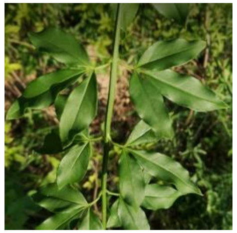	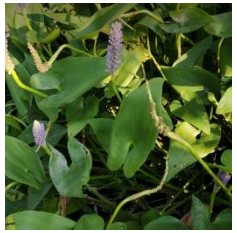	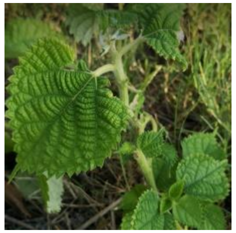
plant038, Phyllostachys sulphurea (Carr.) A. et C. Riv.	plant039, Nandina domestica Thunb.	plant040, Galium odoratum (L.) Scop.	plant041, Sapium sebiferum (L.) Roxb.	plant042, Jasminum nudiflorum Lindl.	plant043, Pontederia cordata L.	plant044, Boehmeria nivea (L.) Gaudich.
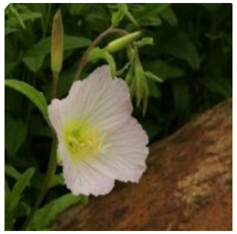	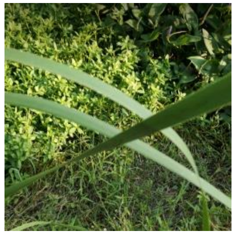	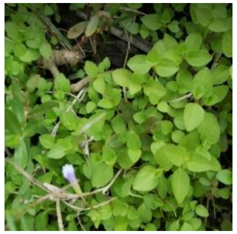	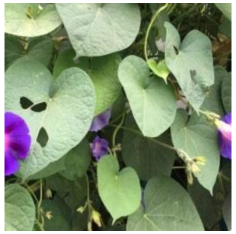	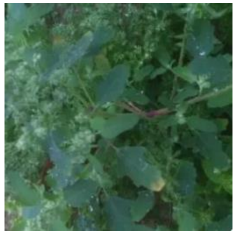	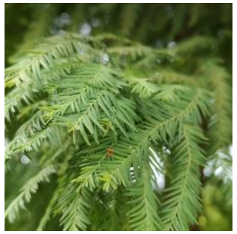	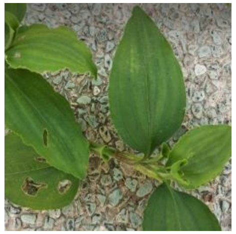

plant045, Oenothera speciosa	plant046, Typha orientalis	plant047, Lindernia crustacea (L.) F. Muell	plant048, Pharbitis nil (L.) Choisy	plant049, Chenopodium album L.	plant050, Taxodium distichum (L.) Rich.	plant051, Commelina bengalensis
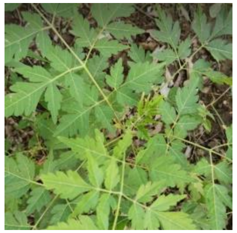	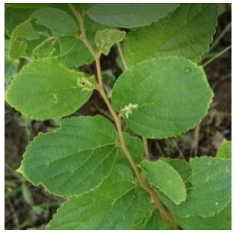	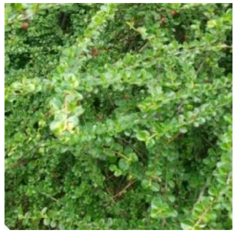	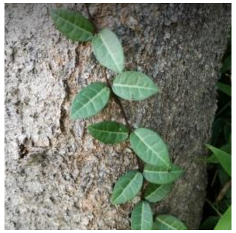	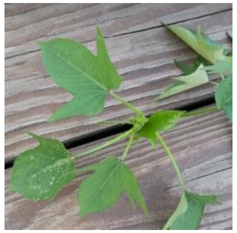	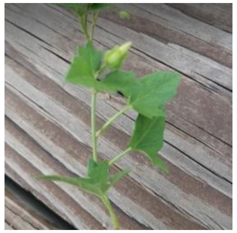	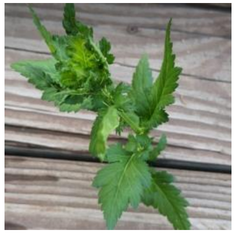
plant052, Melia azedarach L.	plant053, Celtis sinensis Pers.	plant054, Cotoneaster microphyllus Lindl.	plant055, Trachelospermum jasminoides (Lindl.) Lem.	plant056, Ipomoea batatas (L.) Lam.	plant057, Calystegia hederacea Wall.ex.Roxb.	plant058, Dendranthema indicum (L.) Des Moul.
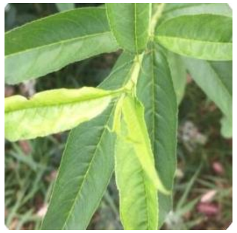	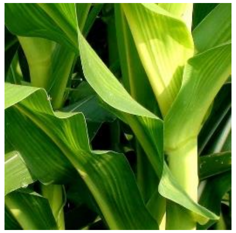	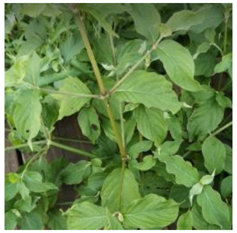	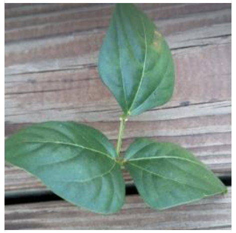	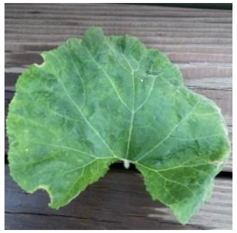	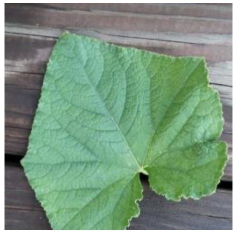	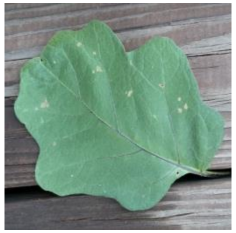
plant059, Amygdalus persica L.	plant060, Zea mays L.	plant061, Achyranthes bidentata Blume	plant062, Vigna unguiculata (Linn.) Walp.	plant063, Cucurbita moschata Duch. ex Poiret	plant064, Cucumis sativus L.	plant065, Solanum melongena L.
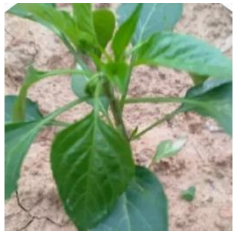	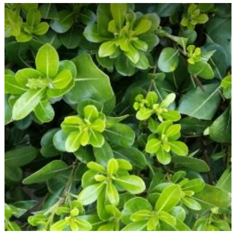	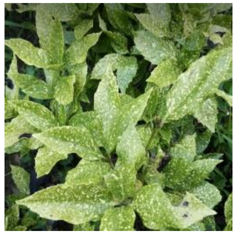	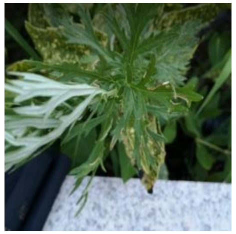	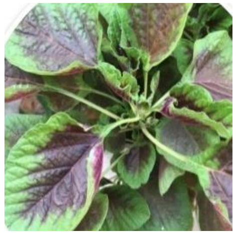	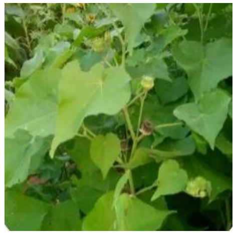	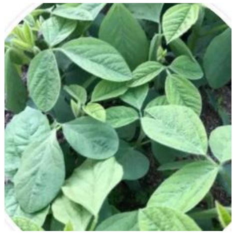

plant066, Capsicum annuum L.	plant067, Pittosporum tobira (Thunb.) Ait.	plant068, Aucuba japonica var. variegata	plant069, Artemisia argyi Levl. et Van.	plant070, Amaranthus tricolor	plant071, Abutilon theophrasti Medicus	plant072, Glycine max (Linn.) Merr.
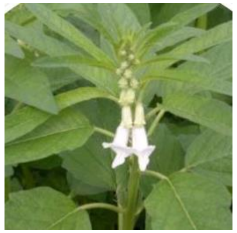	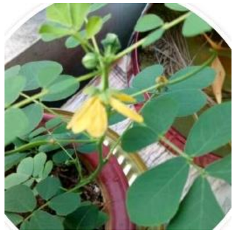	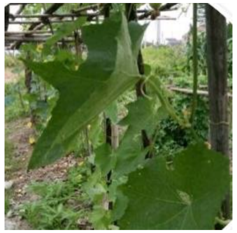	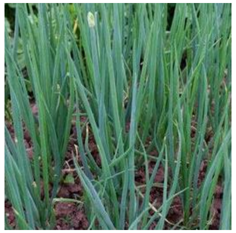	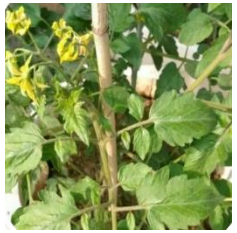	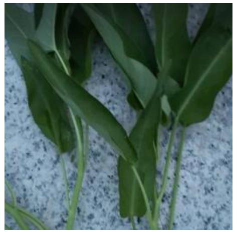	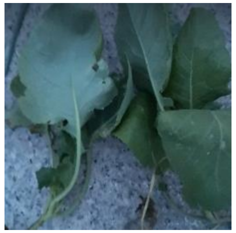
plant073, Sesamum indicum L.	plant074, Cassia tora Linn.	plant075, Luffa cylindrica (L.) Roem.	plant076, Allium fistulosum	plant077, Lycopersicon esculentum Mill.	plant078, Ipomoea aquatica Forsk.	plant079, Brassica caulorapa Pasq.
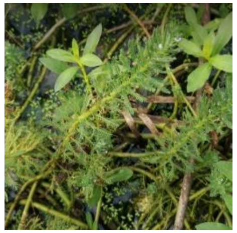	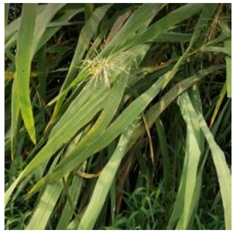	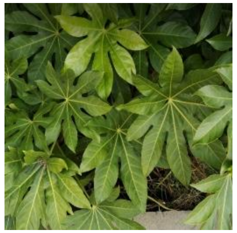	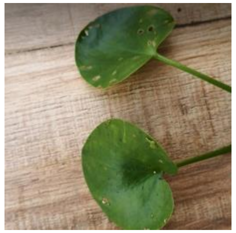	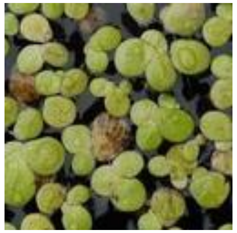	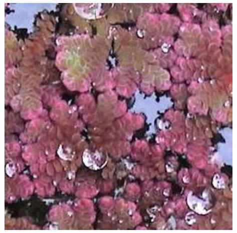	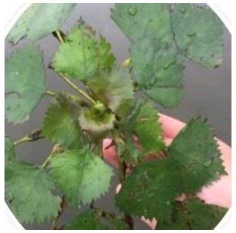
plant080, Myriophyllum verticillatum L.	plant081, Zizania latifolia (Griseb.) Stapf	plant084, Fatsia japonica (Thunb.) Decne. et Planch.	plant085, Hydrocharis dubia	plant086, Spirodela polyrrhiza (L.) Schleid.	plant087, Azolla imbricata (Roxb.) Nakai	plant088, Trapa natans L.
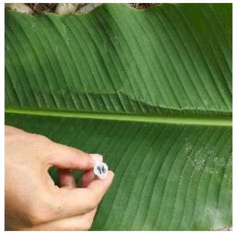	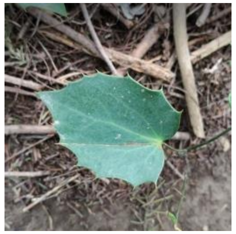	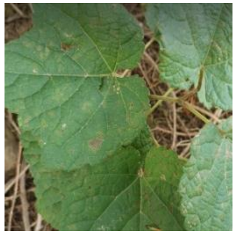	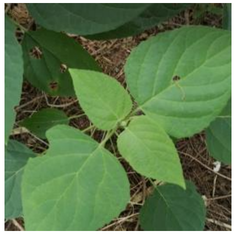	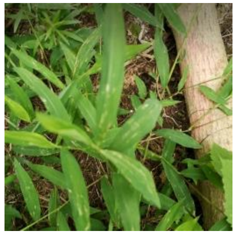	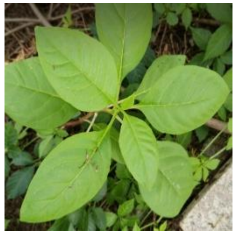	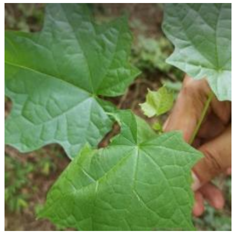

plant096, Canna indica L.	plant097, Mahonia fortunei (Lindl.) Fedde	plant098, Rubus lambertianus Ser.	plant099, Clerodendrum trichotomum Thunb.	plant100, Lophatherum gracile	plant101, Phytolacca acinosa Roxb.	plant102, Alangium chinense (Lour.) Harms
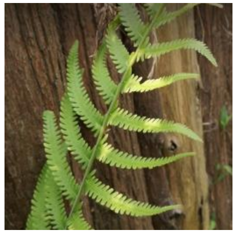	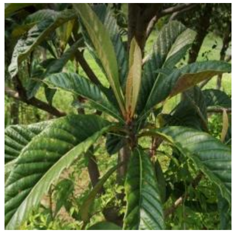	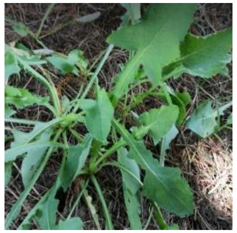	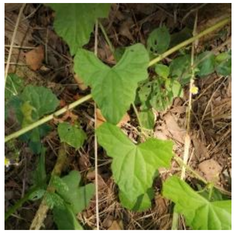	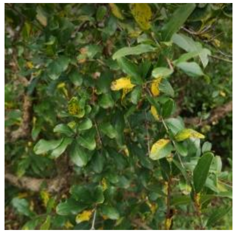	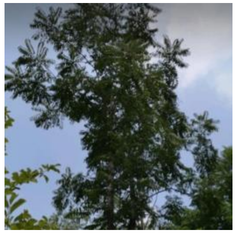	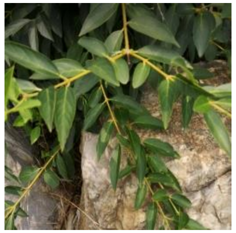
plant103, Cyclosorus interruptus (Willd.) H. Ito	plant104, Eriobotrya japonica (Thunb.) Lindl.	plant106, Sonchus arvensis L.	plant107, Zehneria japonica (Thunberg) H. Y. Liu）	plant108, Punica granatum L.	plant109, Toona sinensis (A. Juss.) Roem.	plant110, Forsythia suspensab (Thunb.) Vahl
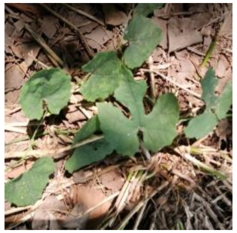	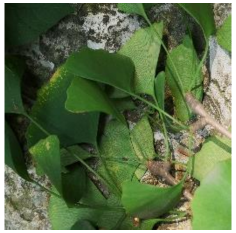	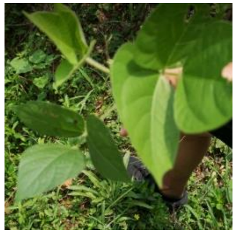	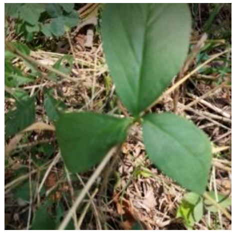	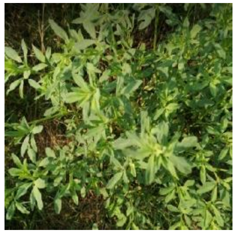	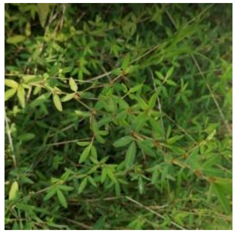	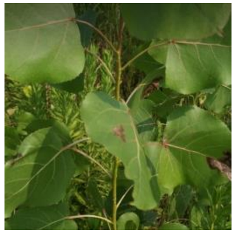
plant111, Trichosanthes kirilowii Maxim.	plant112, Ginkgo biloba L.	plant113, Pueraria lobata (Willd.) Ohwi	plant114, Pinellia ternata	plant118, Melilotus officinalis (L.) Pall.	plant119, Kummerowia striata (Thunb.) Schindl.	plant120, Populus tomentosa
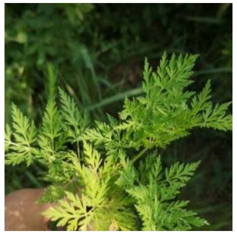	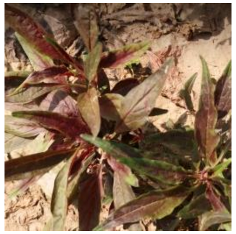	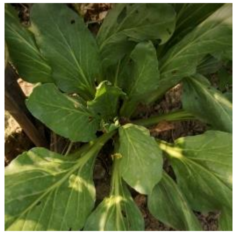	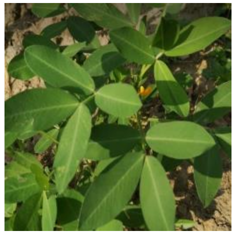	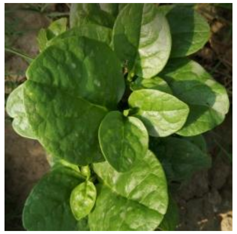	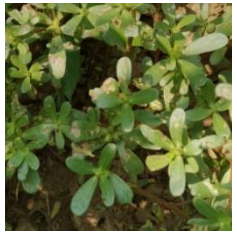	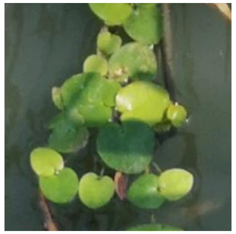

plant121, Cnidium monnieri (L.) Cuss.	plant122, Celosia argentea L.	plant123, Brassica chinensis L.	plant124, Arachis hypogaea Linn.	plant125, Basella alba L.	plant126, Portulaca oleracea L.	plant127, Hydrocotyle vulgaris
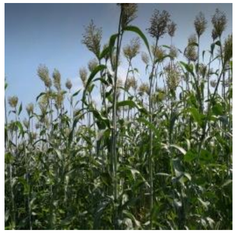	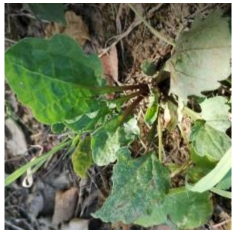	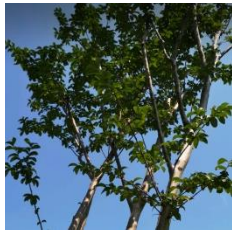	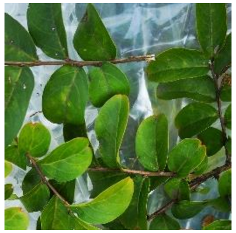	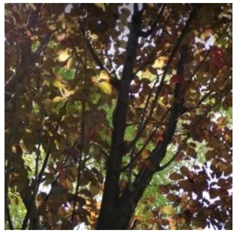	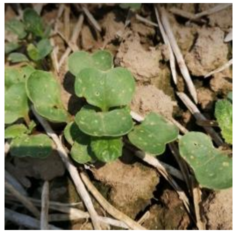	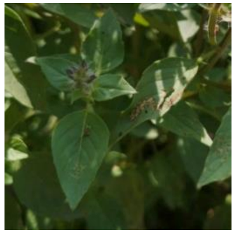
plant128, Sorghum bicolor (L.) Moench	plant129, Gerbera anandria (L.) Sch.-Bip.	plant130, Eucommia ulmoides Oliver	plant131, Lagerstroemia indica L.	plant132, Prunus cerasifera Ehrhar (Jacq.) Rehd.	plant133, Raphanus sativus L.	plant134, Ocimum basilicum L.
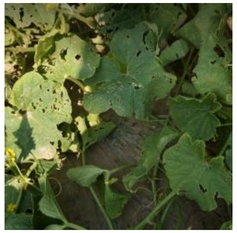	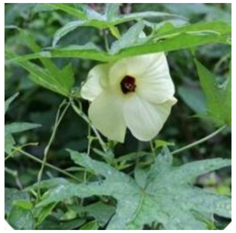	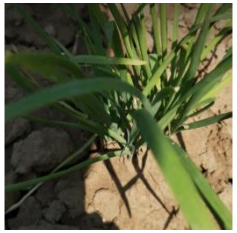	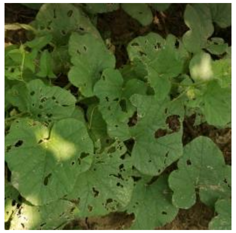	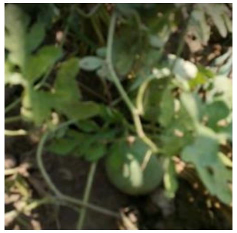	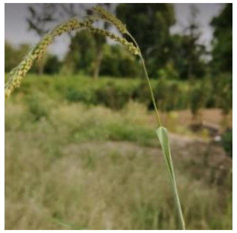	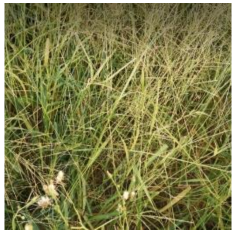
plant135, Cucumis melo L.	plant136, Abelmoschus manihot (Linn.) Medicus	plant137, Allium tuberosum	plant139, Cucumis melo L. var. agrestis Naud.	plant140, Citrullus lanatus (Thunb.) Matsum. et Nakai	plant142, Echinochloa crusgalli (L.) Beauv.	plant143, Digitaria sanguinalis (L.) Scop.
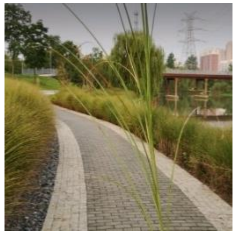	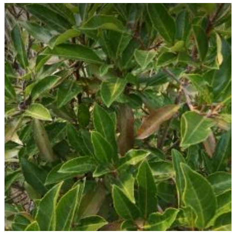	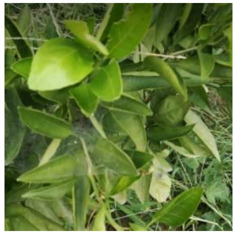	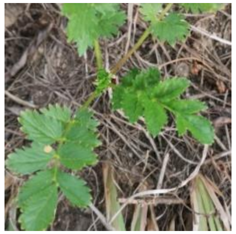	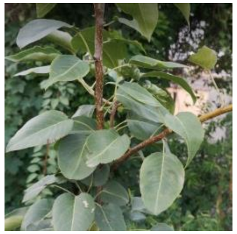	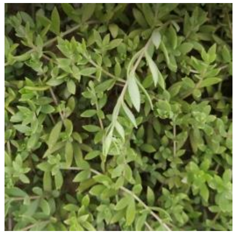	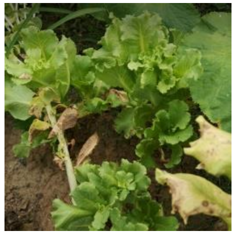

plant144, Miscanthus sinensis Anderss.	plant146, Viburnum Odoratissimum Ker-Gawl.	plant147, Citrus reticulata Blanco Blanco	plant148, Potentilla supina L.	plant149, Pyrus communis	plant150, Sedum sarmentosum Bunge	plant151, Lactuca sativa L. var. ramosa Hort.
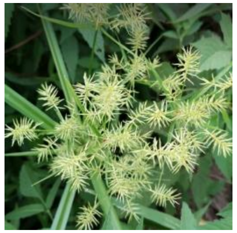	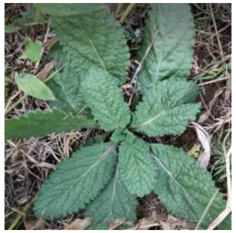	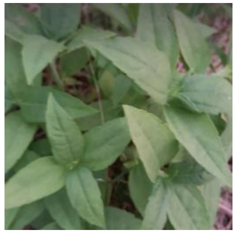	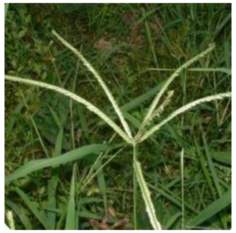	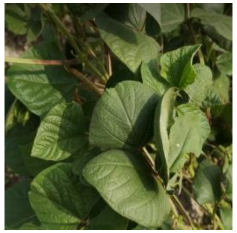	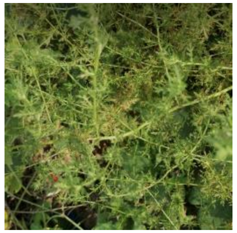	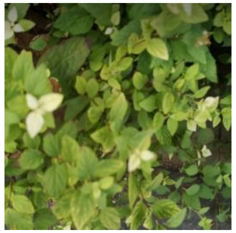
plant153, Cyperus rotundus L.	plant155, Salvia plebeia R. Br.	plant156, Eclipta prostrata (L.) L.	plant157, Eleusine indica (L.) Gaertn.	plant158, Vigna radiata (Linn.) Wilczek	plant159, Artemisia carvifolia	plant160, Spiraea salicifolia
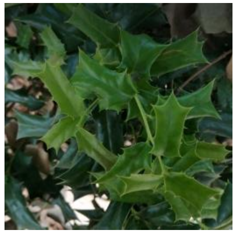	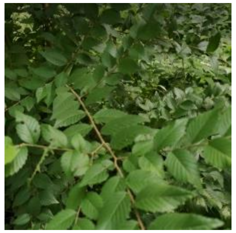	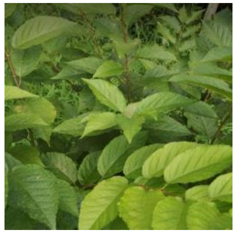	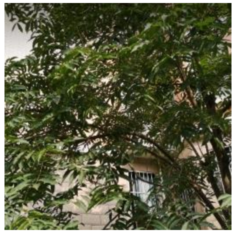	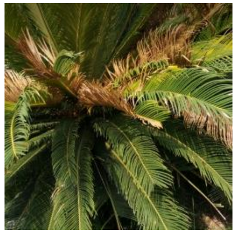	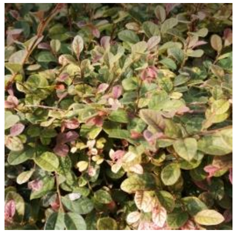	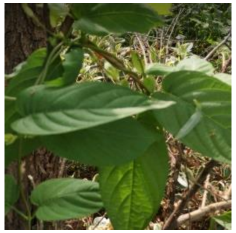
plant161, Ilex cornuta Lindl. et Paxt.	plant162, Ulmus pumila L.	plant163, Cerasus yedoensis (Matsum.) Yu et Li	plant164, Sapindus mukorossi Gaertn.	plant165, Cycas revoluta Thunb.	plant166, Loropetalum chinense var.rubrum Yieh	plant167, Paederia foetida
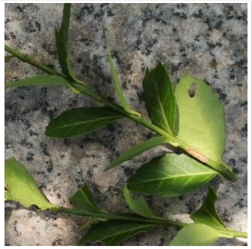	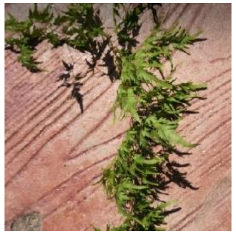	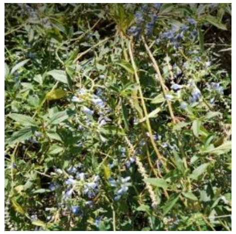	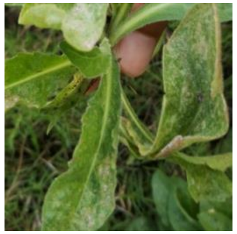	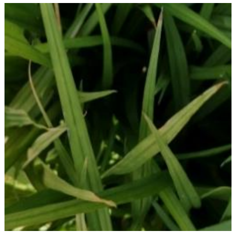	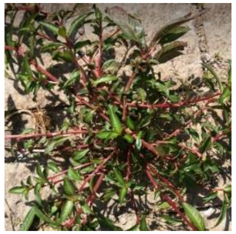	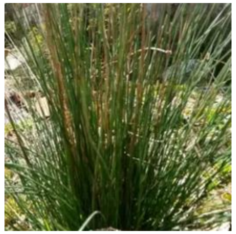

plant168, Euonymus alatus (Thunb.) Sieb.	plant169, Lygodium japonicum (Thunb.) Sw.	plant170, Salvia japonica Thunb.	plant171, Elephantopus scaber L.	plant172, Reineckia carnea (Andr.) Kunth	plant173, Ludwigia prostrata Roxb.	plant174, Juncus effusus L.
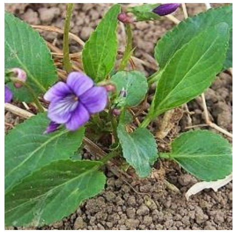	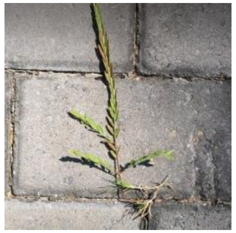	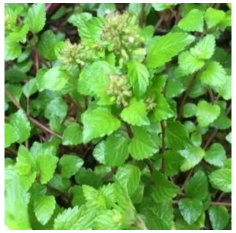	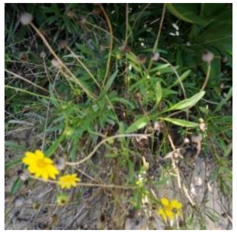	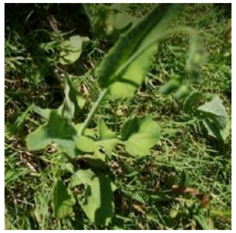	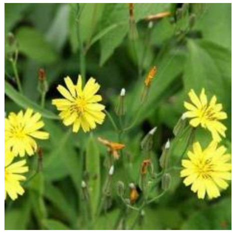	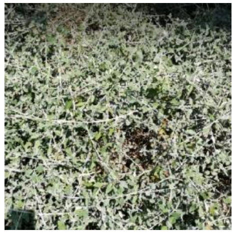
plant175, Viola philippica	plant176, Phyllanthus urinaria L.	plant177, Clinopodium chinense (Benth.) O. Ktze.	plant178, Coreopsis drummondii Torr. et Gray	plant179, Emilia sonchifolia (L.) DC.	plant180, Sonchus oleraceus L.	plant181, Teucrium fruticans
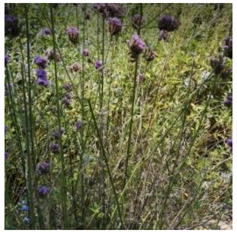	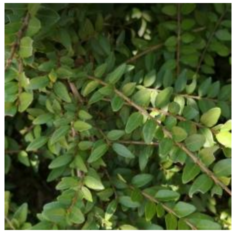	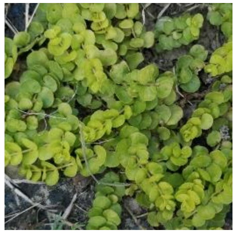	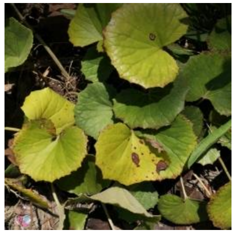	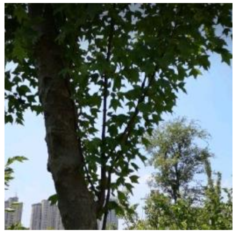	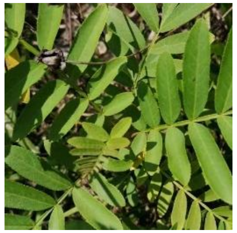	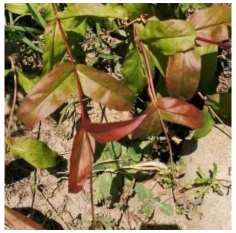
plant182, Verbena officinalis L.	plant183, Lonicera japonica	plant184, Lysimachia christinae Hance	plant185, Centella asiatica (L.) Urban	plant186, Acer buergerianum Miq.	plant187, Pterocarya stenoptera	plant188, Hypericum monogynum L.
