# Supplementary material for: Expanding known viral diversity in plants: virome of 161 species alongside an ancient canal
Source: Environ Microbiome. 2022 Nov 27;17:58. doi: 10.1186/s40793-022-00453-x (PMC9703751; doi:10.1186/s40793-022-00453-x)

Supplementary Figure 2. The map for sampling site of plant samples in this study. The numbers on the map show the sampling sites where the corresponding numbers of plant sample were collected.

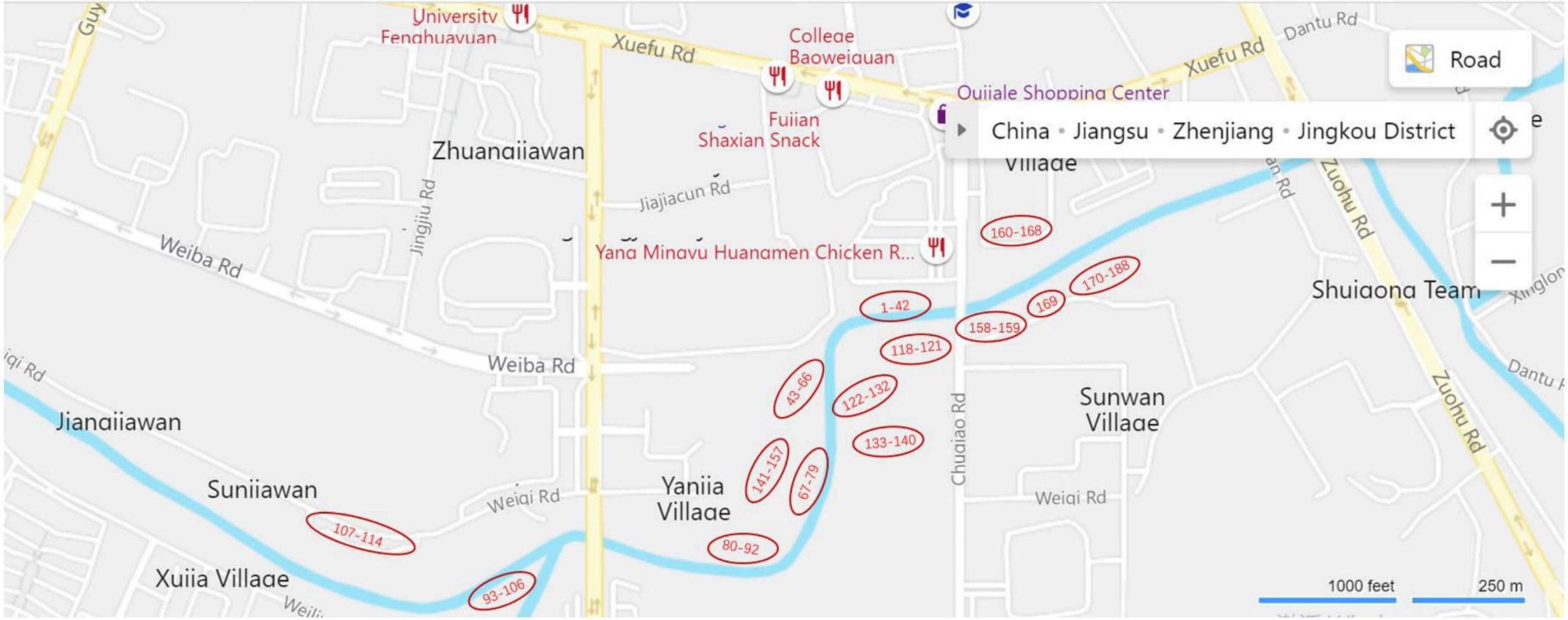

Supplementary Figure 3. Phylogenetic tree of plant-associated viruses belonging in the family *Dicistroviridae*. Bayesian inference trees were constructed using MrBayes v3.2 based on amino acid sequence of RdRp domain of RNA viruses, NS protein of parvovirus-like viruses, Rep protein of CRESS DNA virus, or Major capsid protein of microvirus. The names of virus strains which were newly identified are shadowed with different colors based on their host species. The silhouetted organism denotes host taxa of viruses identified in the present study and their best matched relatives based on BLASTx searching in GenBank. Virus family or genus within which the certain cluster of viruses belong is shown. Red dots represent 100% posterior probability support for MrBayes.

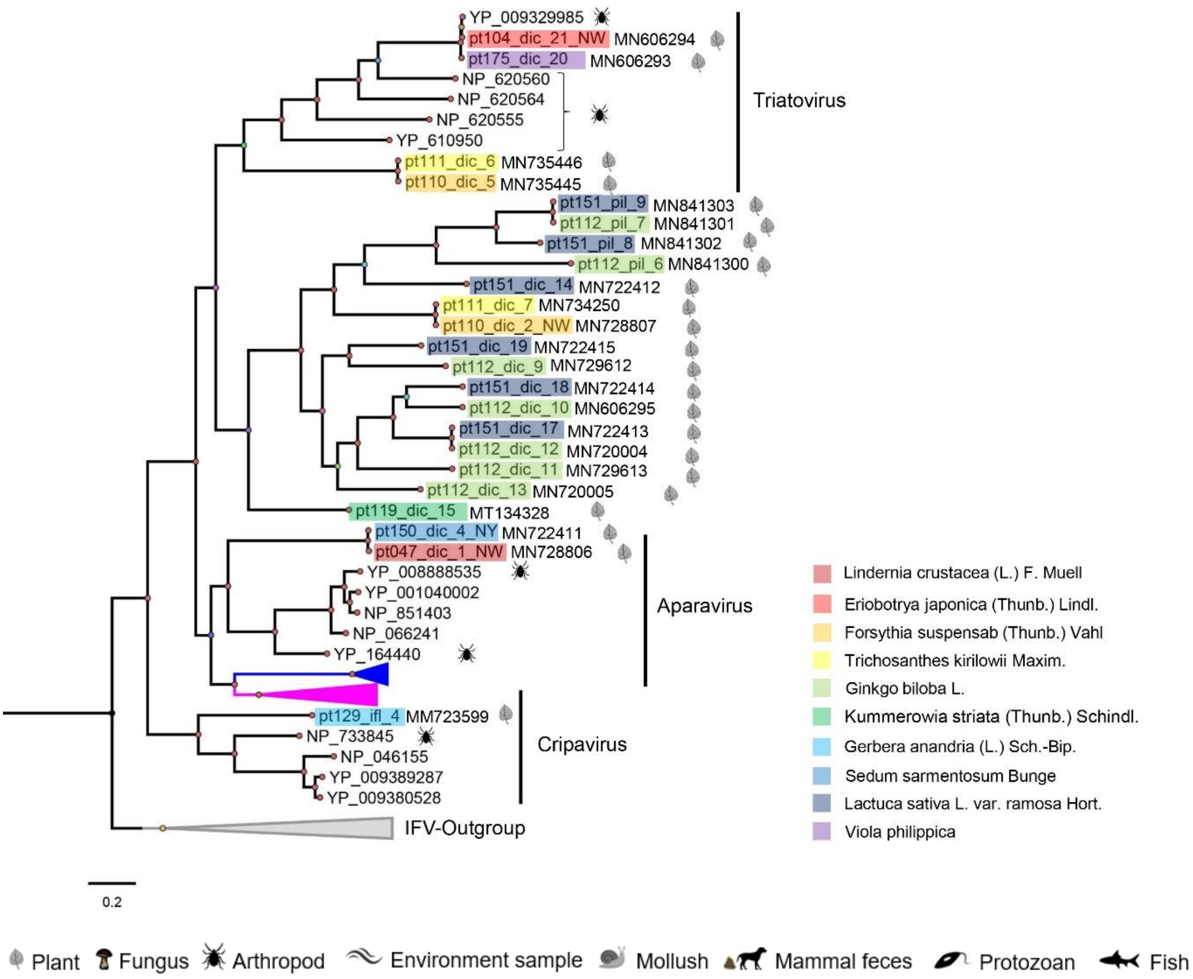

Supplementary Figure 4. Viral genome organizations of plant-associated viruses belonging in the family *Dicistroviridae*. The genome structure of the reference strain and those viruses identified in this study are drawn. Within each genome, the upper gray boxes are ORFs, while the underneath boxes define regions with blast matches to a viral protein or a protein domain whose detailed information is provided under each box. Different protein or domain is marked with different color.

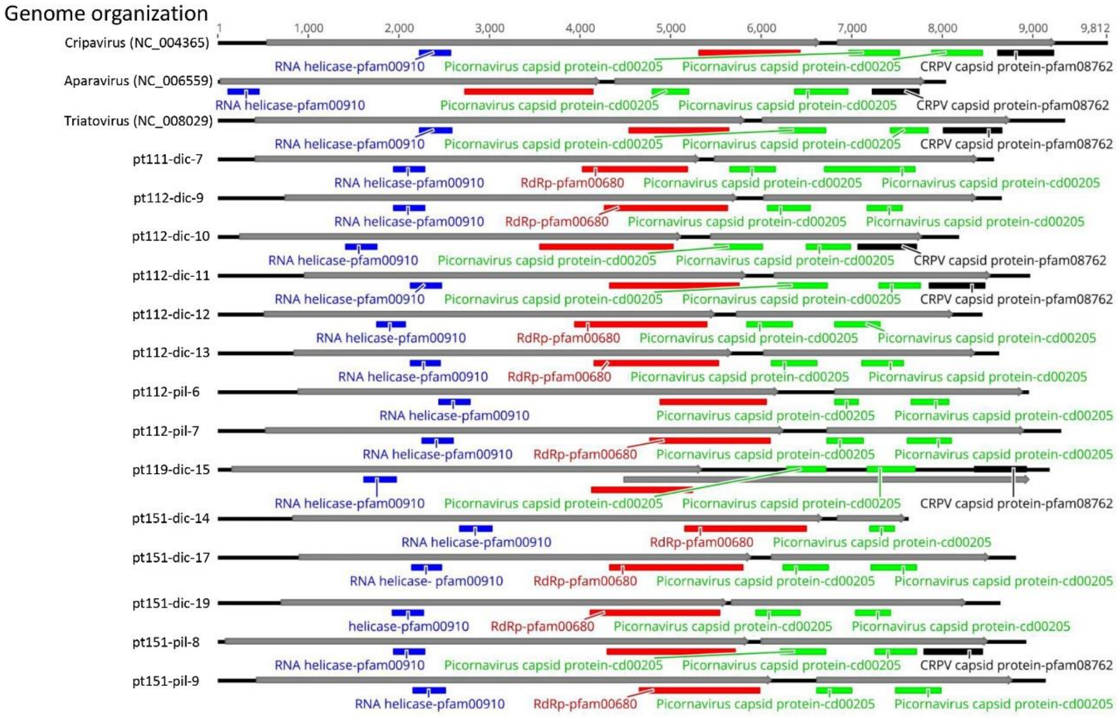

Supplementary Figure 5. Phylogenetic tree of plant-associated viruses belonging in the family *Iflaviridae*. Figure legend is the same as Supplementary Figure 3.

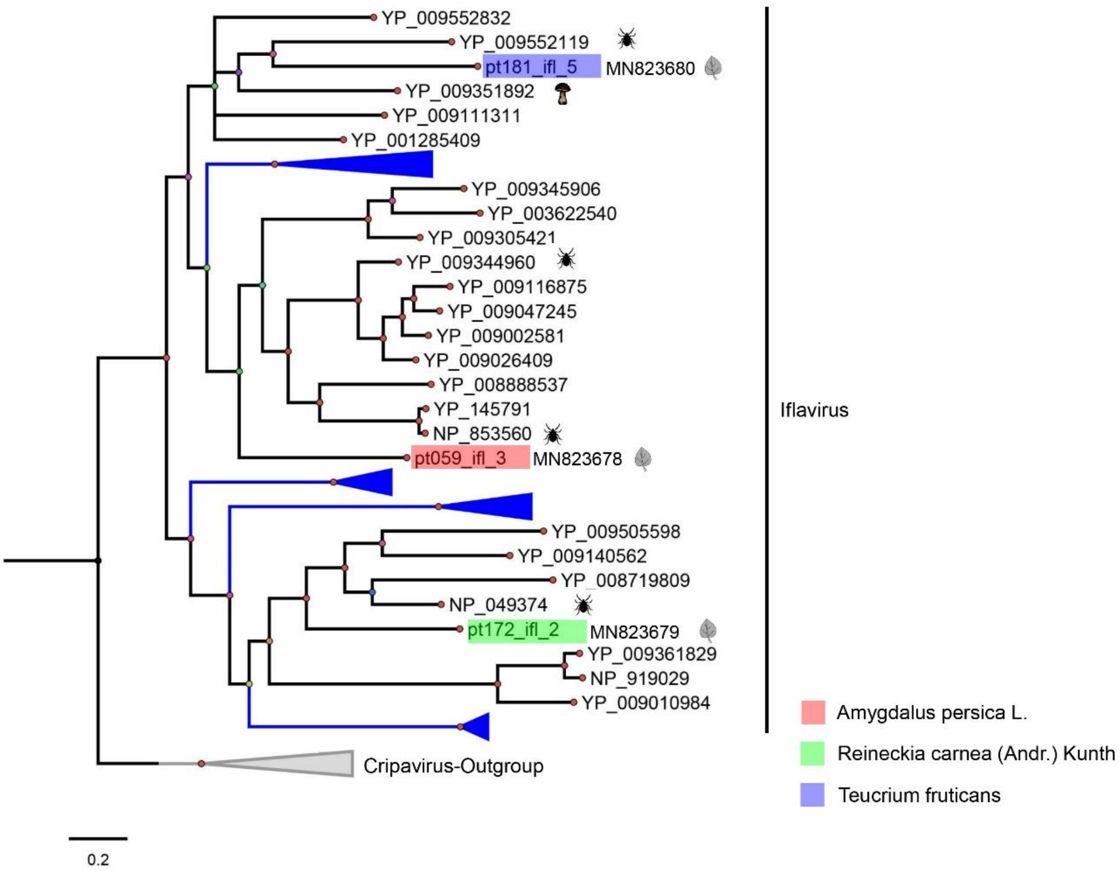

Supplementary Figure 6. Viral genome organizations of plant-associated viruses belonging in the family *Marnaviridae*. Figure legend follows Supplementary Figure 4.

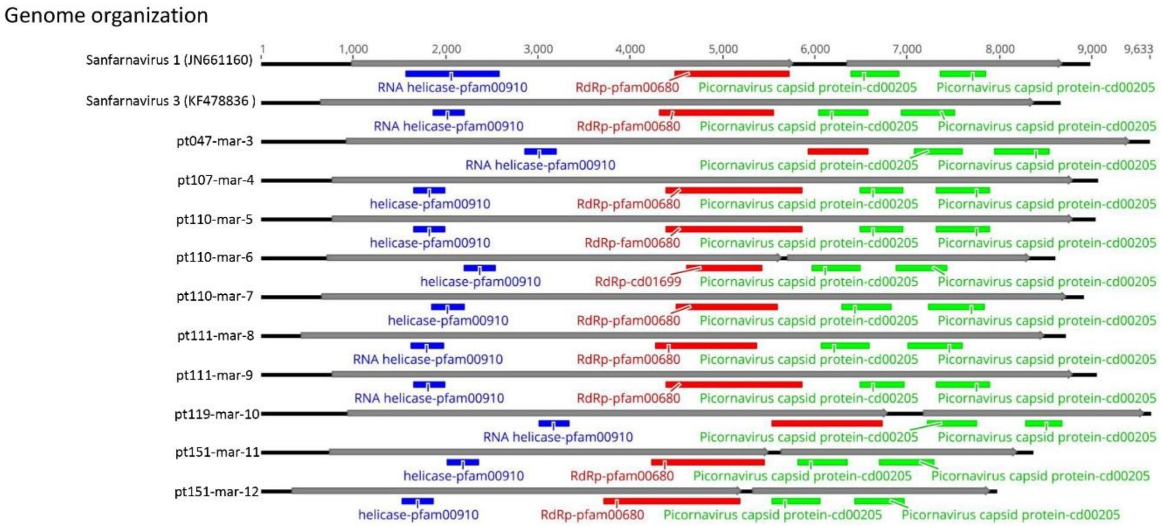

Supplementary Figure 7. The phylogenetic tree of plant-associated viruses belonging in the family *Marnaviridae*. Figure legend is the same as Supplementary Figure 3.

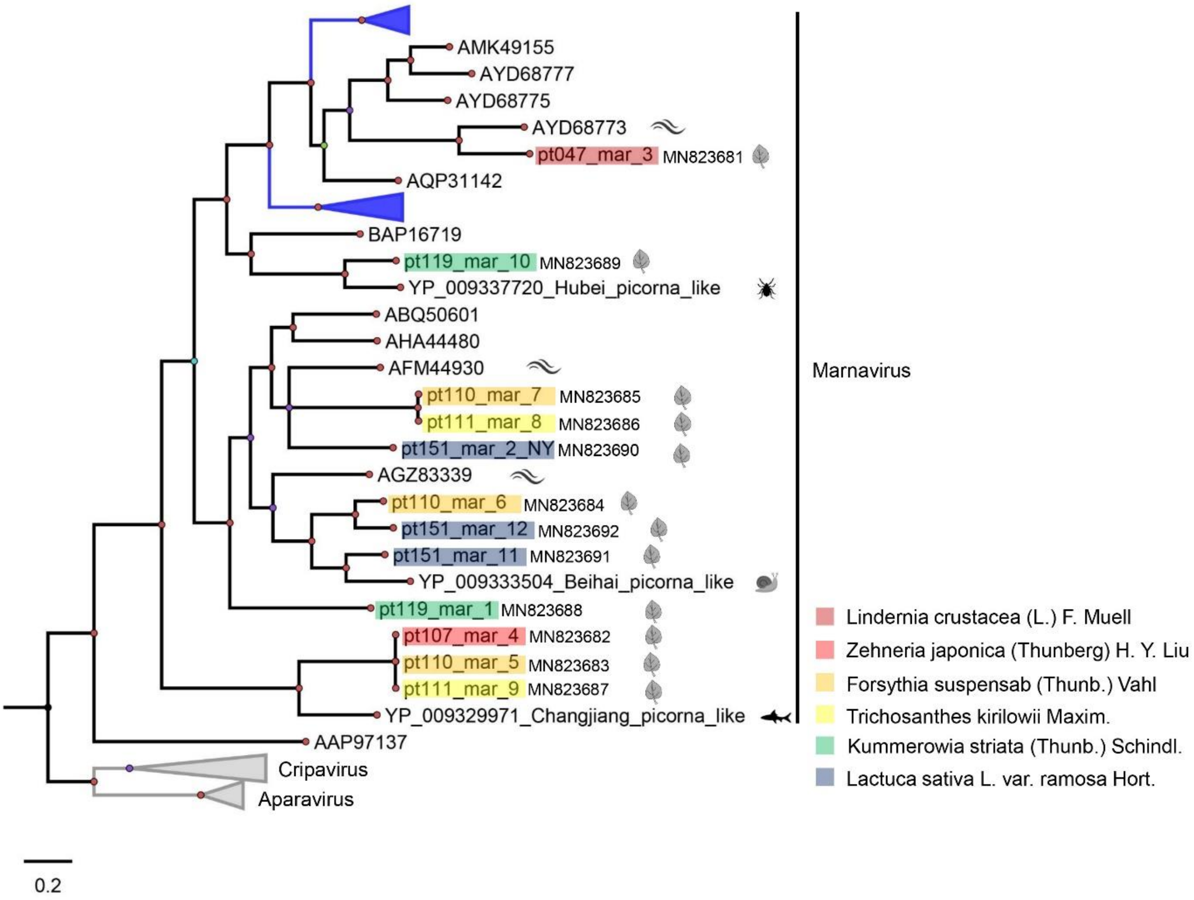

Supplementary Figure 8. The phylogenetic tree and viral genome organization of plant-associated Picorna-like viruses. Figure legend is the same as Supplementary Figure 3 and 4.

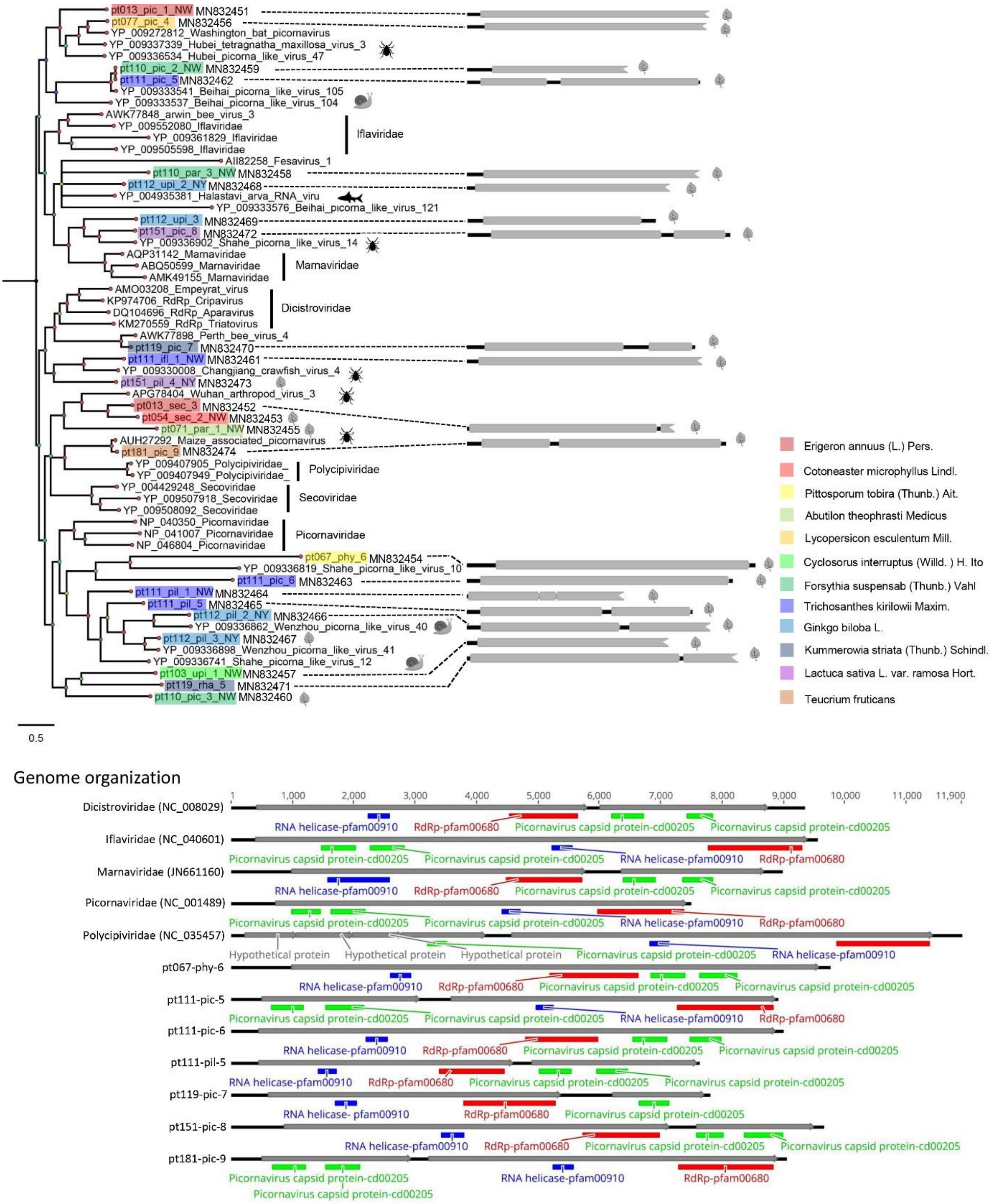

Supplementary Figure 9. The phylogenetic tree of plant-associated Noda-like viruses. Figure legend is the same as Supplementary Figure 3.

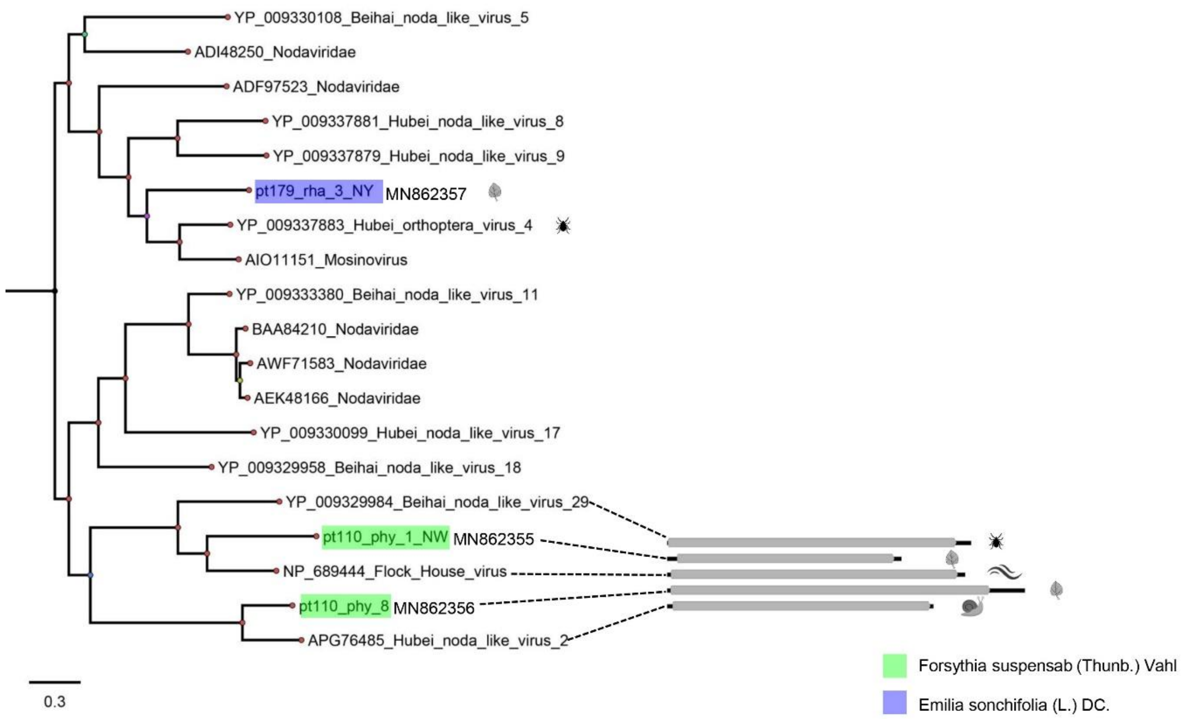

Supplementary Figure 10. The phylogenetic tree of plant-associated Permutotetra-like viruses. Figure legend is the same as Supplementary Figure 3.

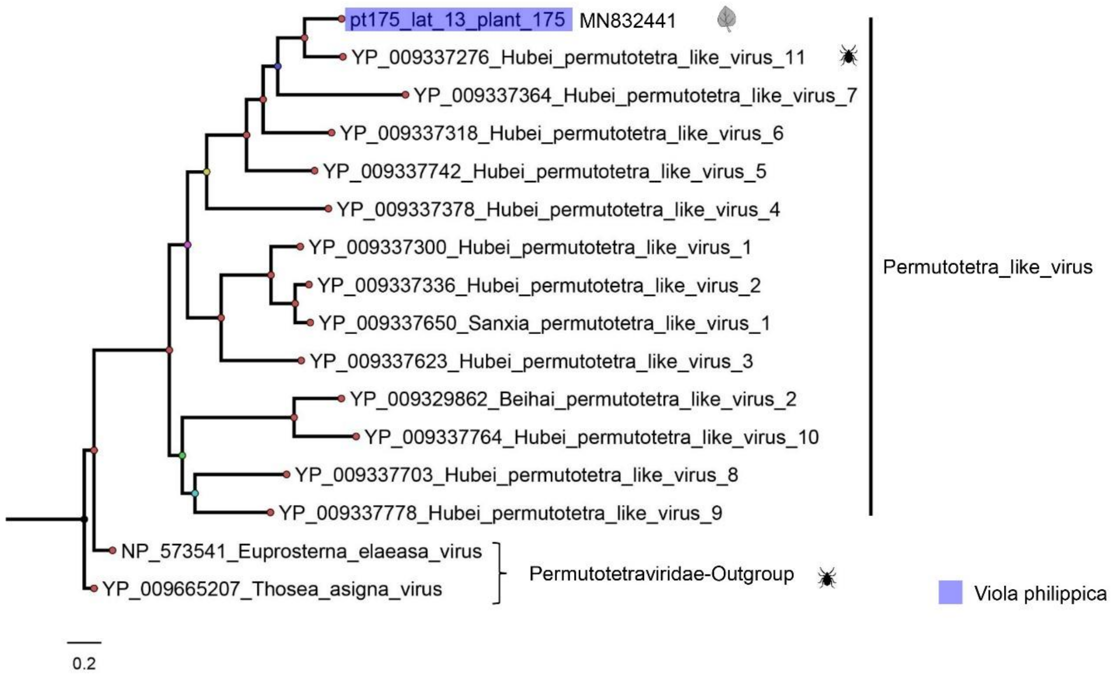

Supplementary Figure 11. The phylogenetic tree of plant-associated Yanvirus-like viruses. Figure legend is the same as Supplementary Figure 3.

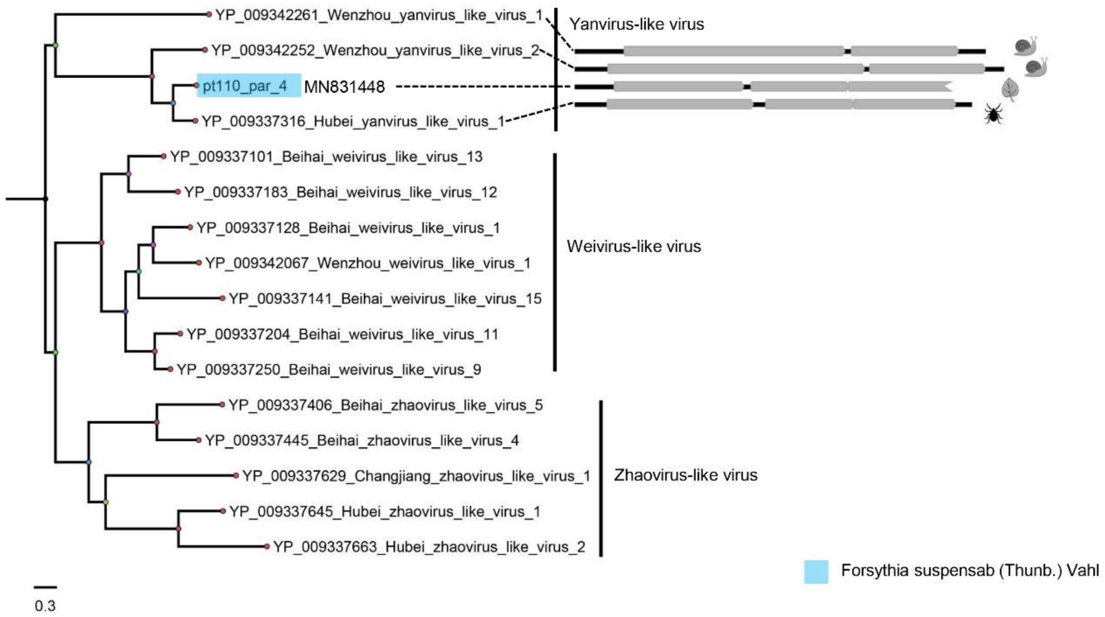

Supplementary Figure 12. The phylogenetic tree and viral genome organization of plant-associated Chuvirus-like viruses. Figure legend is the same as Supplementary Figure 3 and 4.

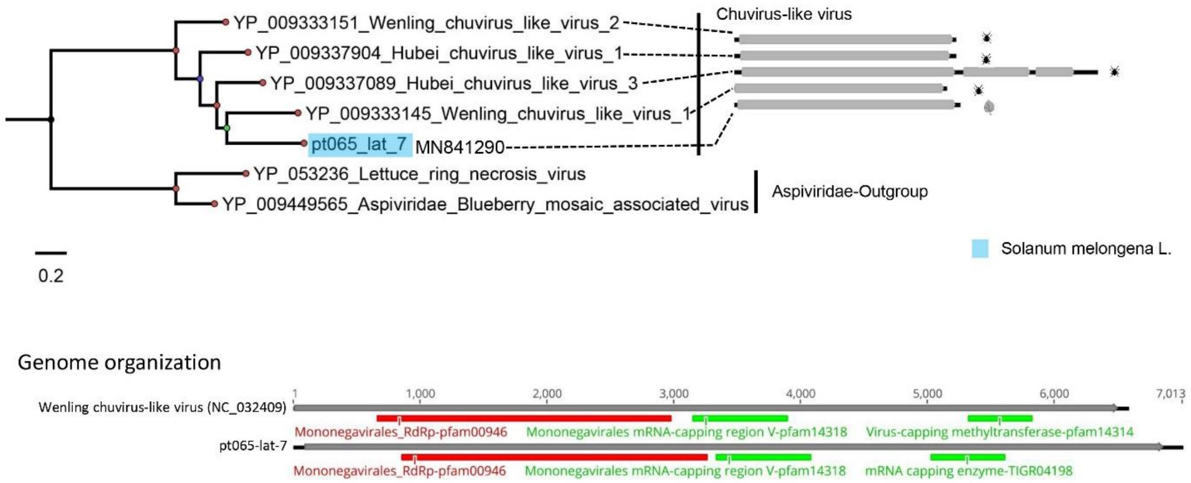

Supplementary Figure 13. The phylogenetic tree of plant-associated parvovirus-like viruses. Figure legend is same as Supplementary Figure 3.

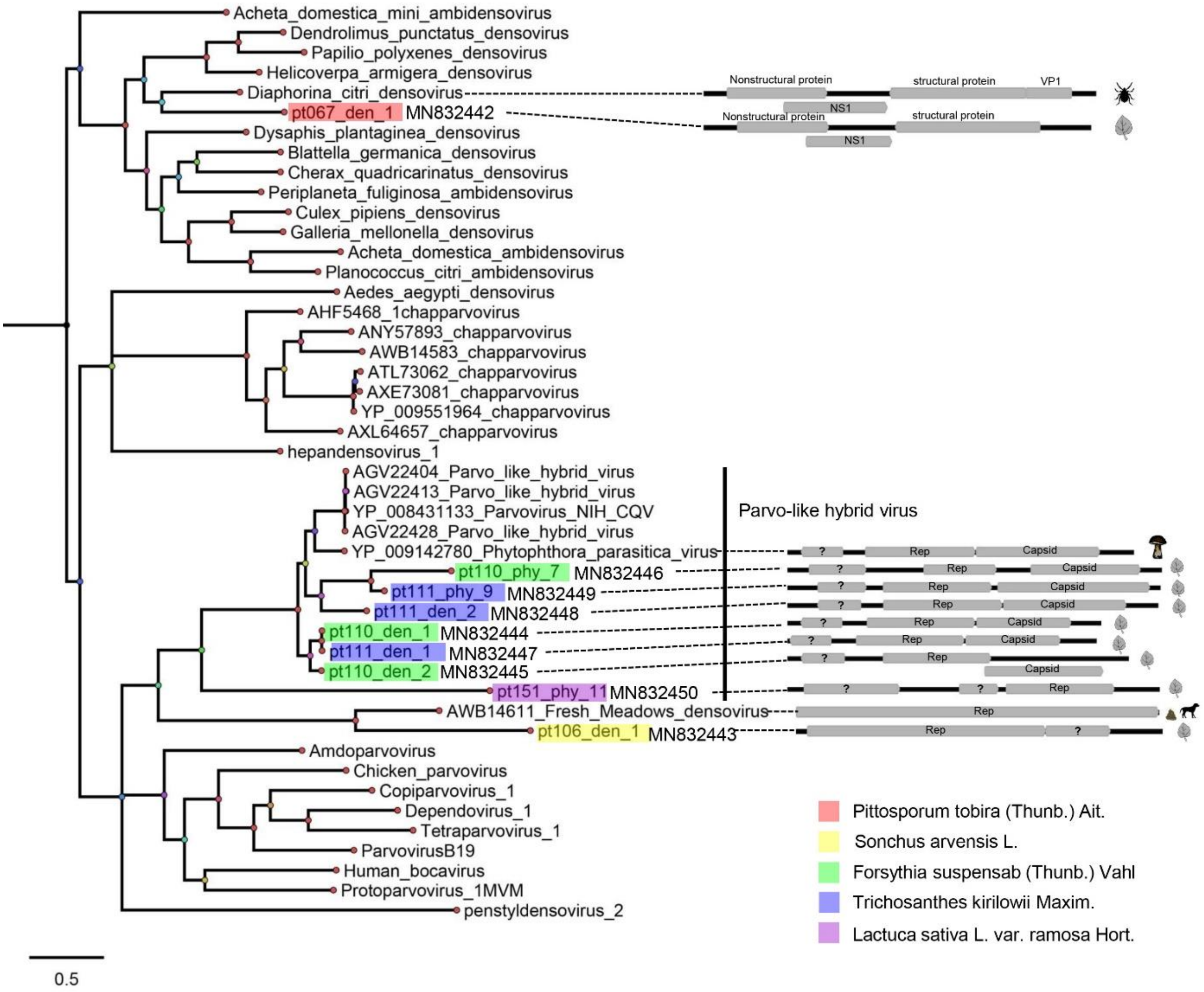

Supplementary Figure 14. Viral genome organizations of the plant-associated parvovirus-like viruses. Figure legend follows Supplementary Figure 4.

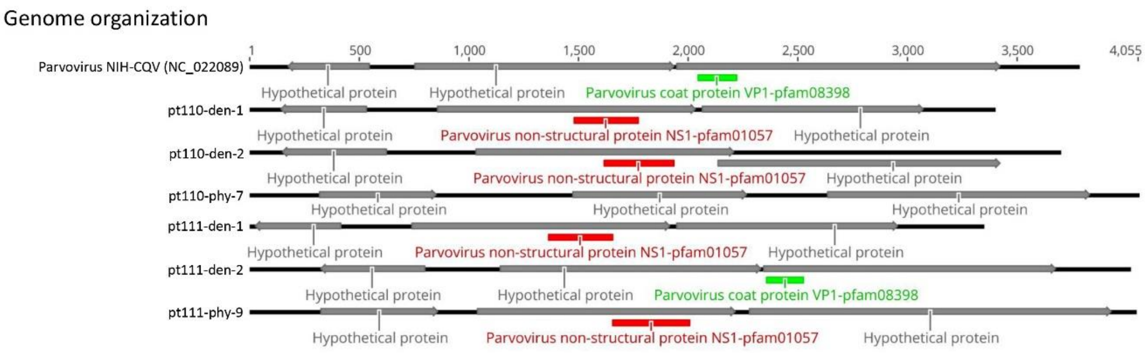

Supplementary Figure 15. The phylogenetic tree and viral genome organization of plant-associated Bastro-like viruses. Figure legend is the same as Supplementary Figure 3 and 4.

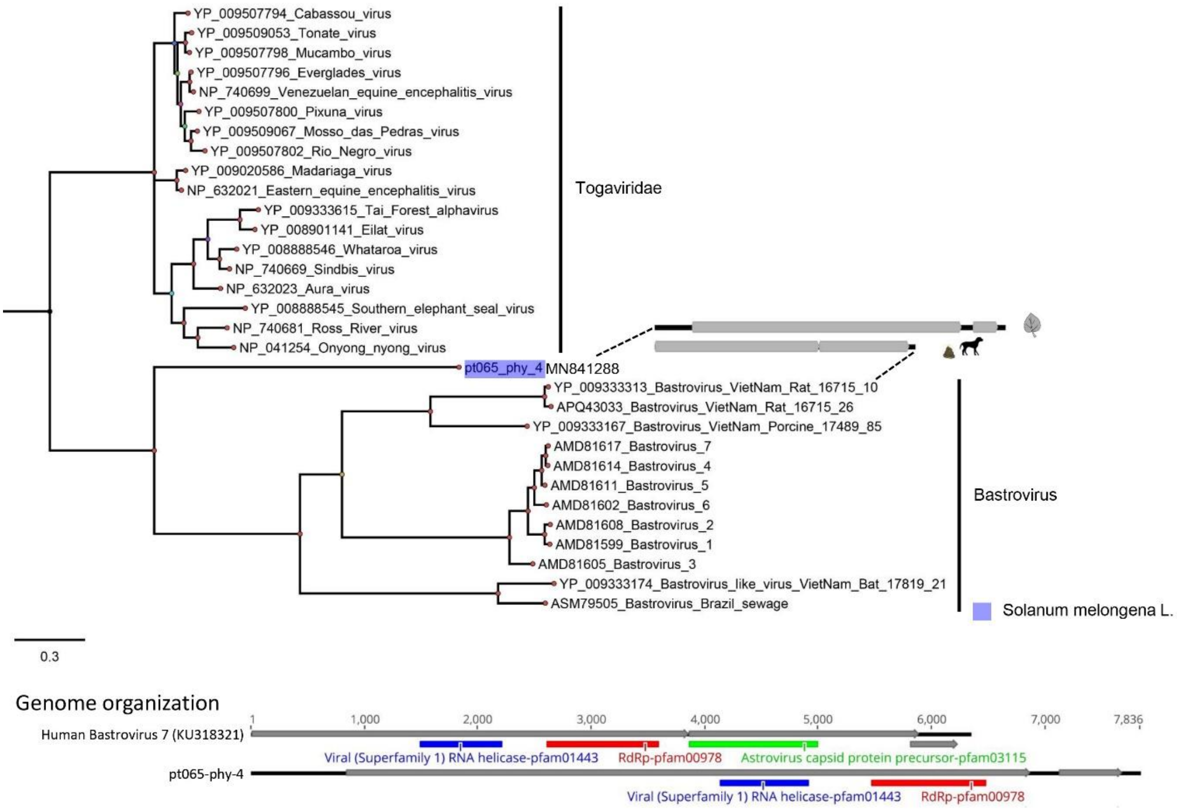

Supplementary Figure 16. The phylogenetic tree and viral genome organization of plant-associated Hepe-like viruses. Figure legend is the same as Supplementary Figure 3 and 4.

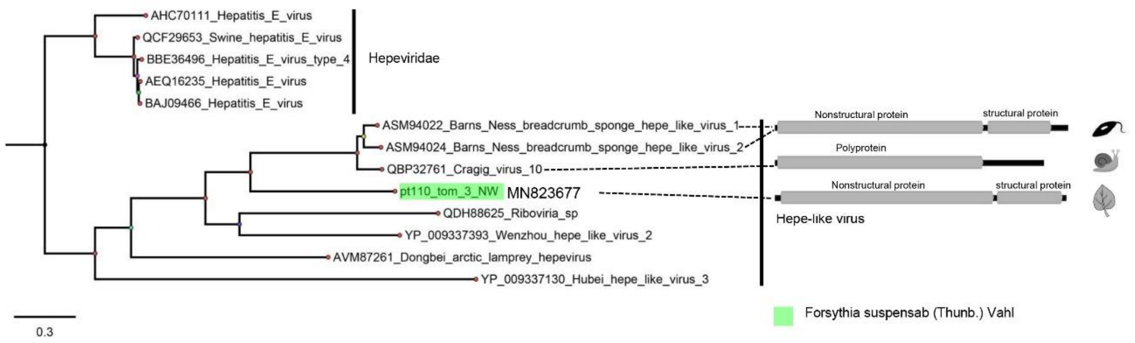

Genome organization

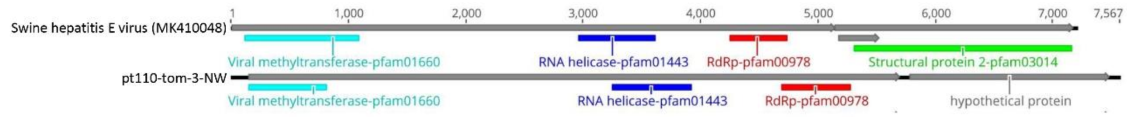

Supplementary Figure 17. The phylogenetic tree of plant-associated *Botybirnavirus* viruses. Figure legend is the same as Supplementary Figure 3.

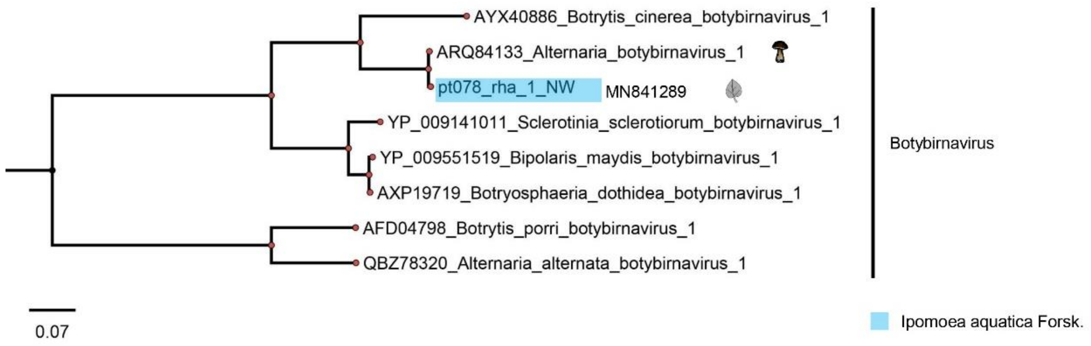

Supplementary Figure 18. The phylogenetic tree and viral genome organization of plant-associated Narna-like viruses. Figure legend is the same as Supplementary Figure 3 and 4.

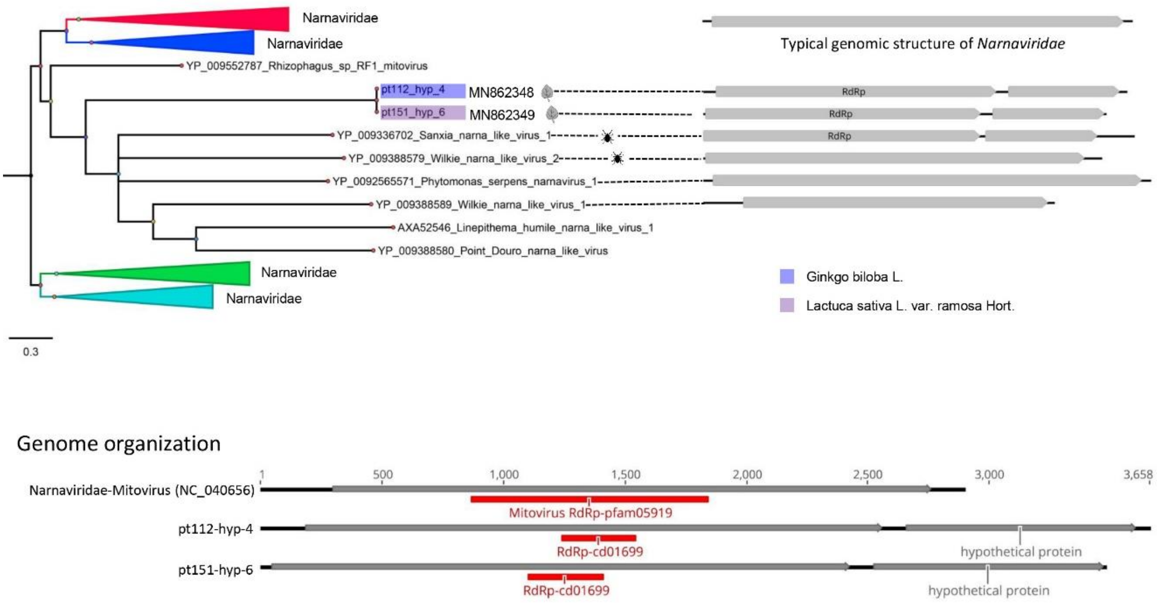

Supplementary Figure 19. The phylogenetic tree of plant-associated viruses belonging in the family *Microviridae*. Figure legend is the same as Supplementary Figure 3.

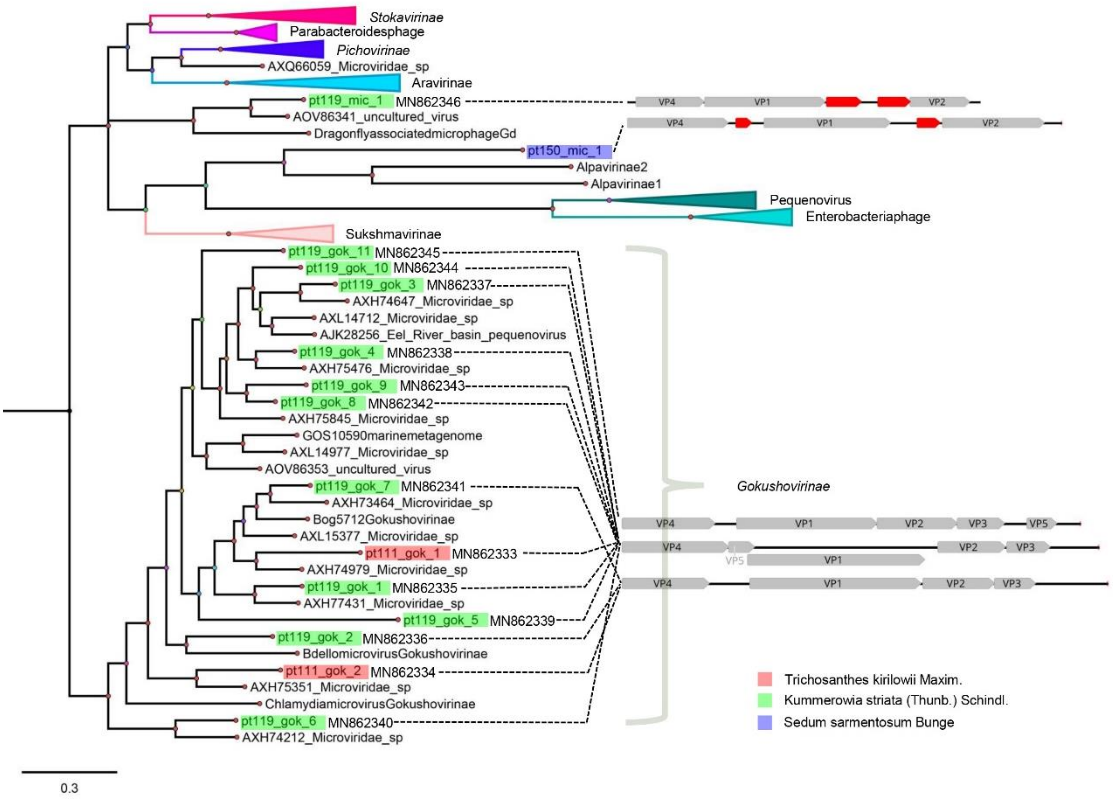

Supplementary Figure 20. The phylogenetic tree of *Potyviridae*. Figure legend is the same as Supplementary Figure 3.

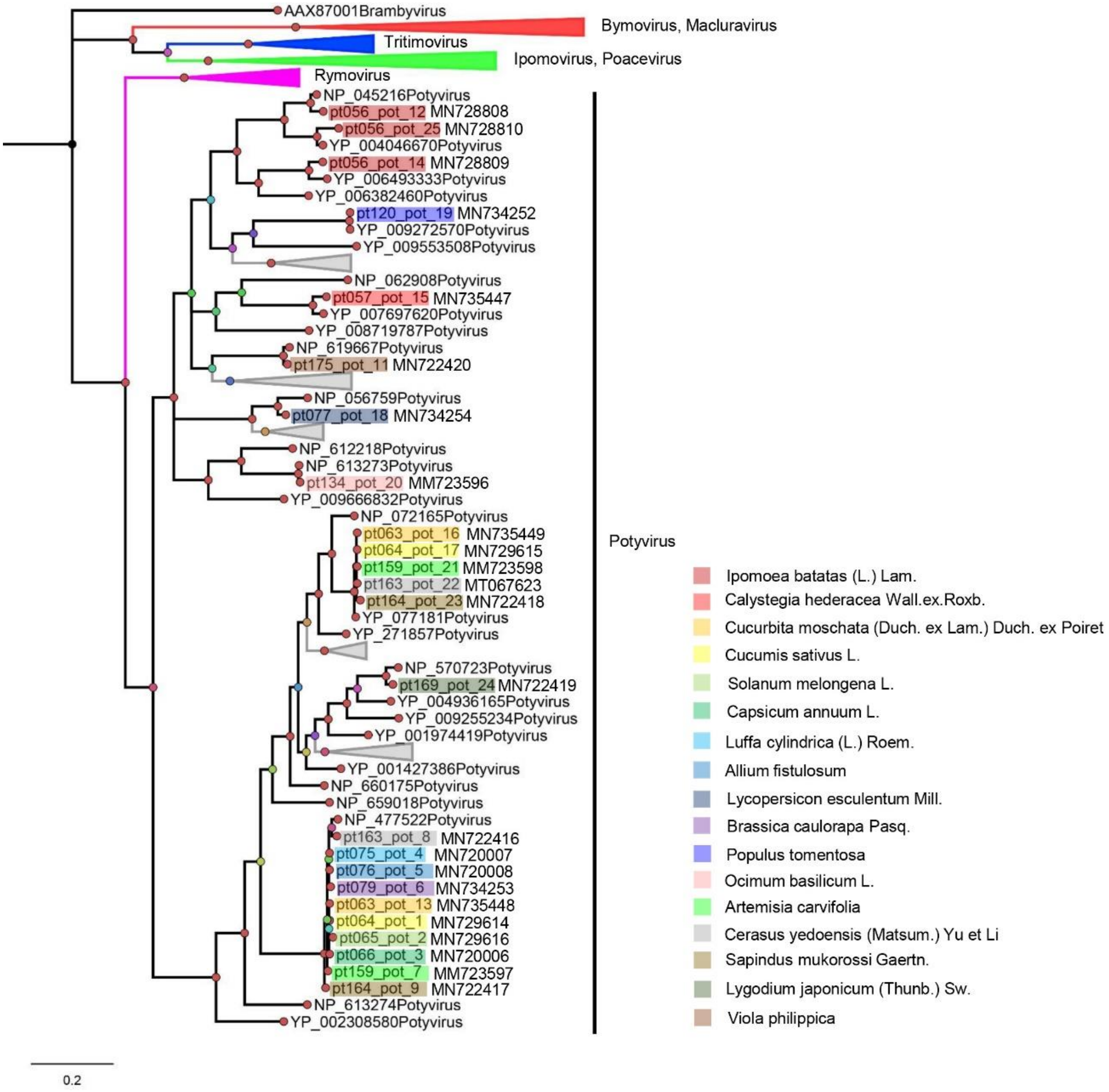

Supplementary Figure 21. The phylogenetic tree of plant viruses belonging in the family *Bromoviridae*. Figure legend is the same as Supplementary Figure 3.

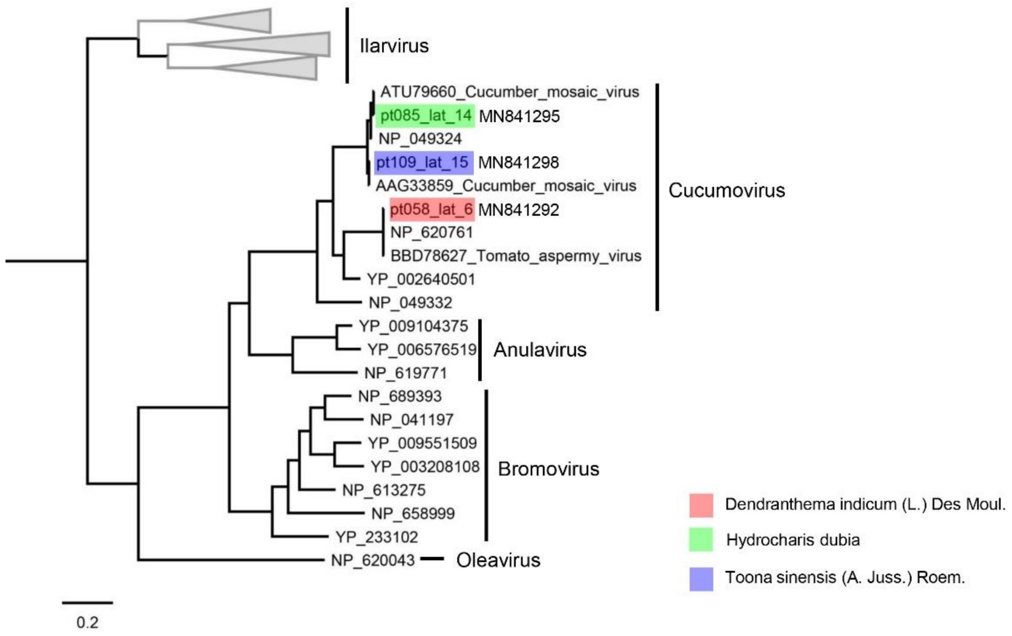

Supplementary Figure 22. The phylogenetic tree of plant viruses belonging in the family *Closteroviridae*. Figure legend is the same as Supplementary Figure 3.

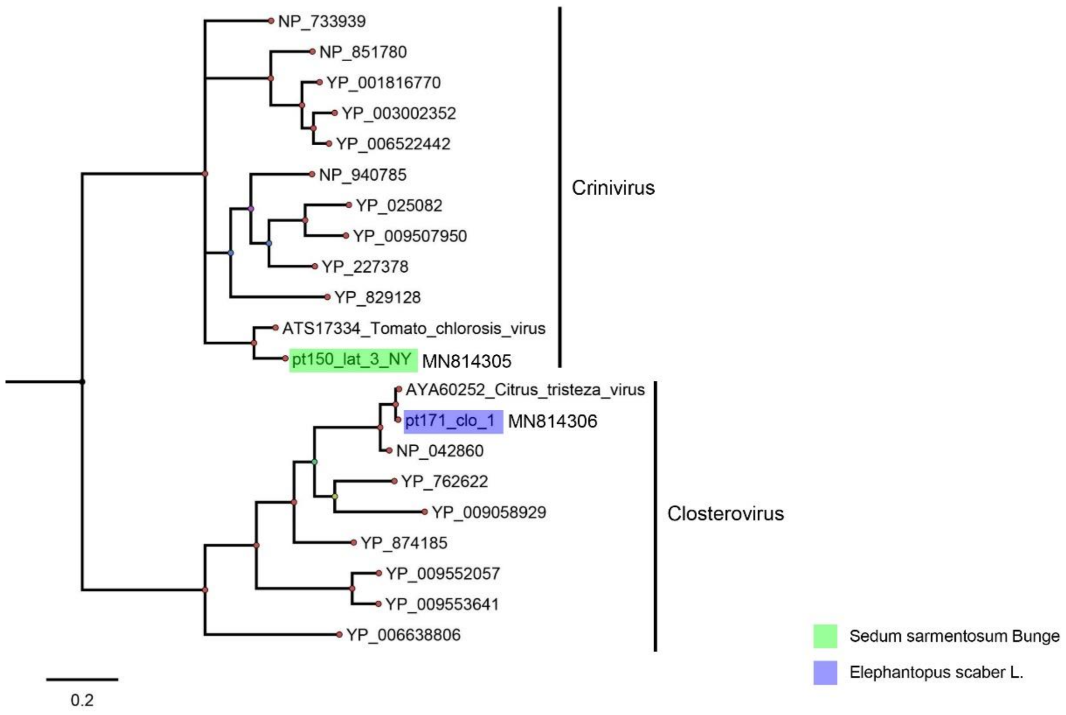

Supplementary Figure 23. The phylogenetic tree of plant viruses belonging in the family *Comovirinae*. Figure legend is the same as Supplementary Figure 3.

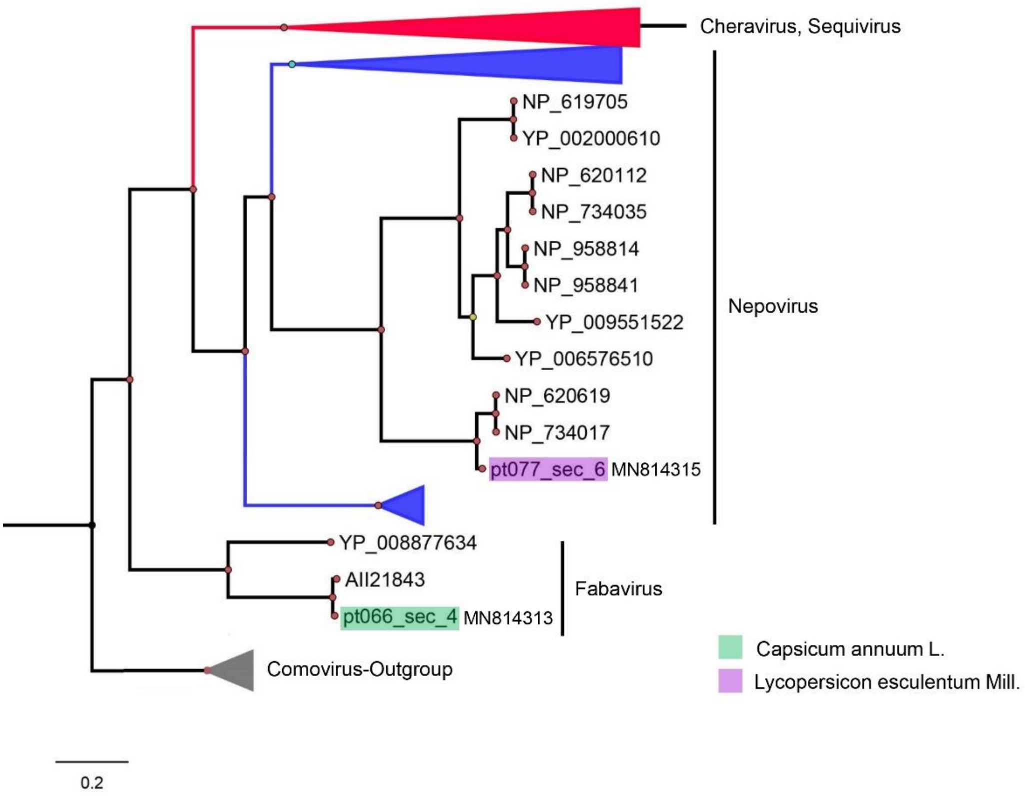

Supplementary Figure 24. The phylogenetic tree of plant viruses belonging in the family *Botourmiaviridae*. Figure legend is the same as Supplementary Figure 3.

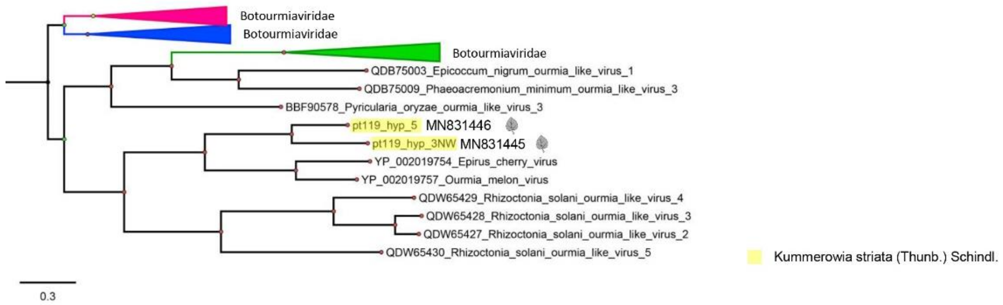

Supplementary Figure 25. The phylogenetic tree of plant viruses belonging in the family *Tymovirales* viruses. Figure legend is the same as Supplementary Figure 3.

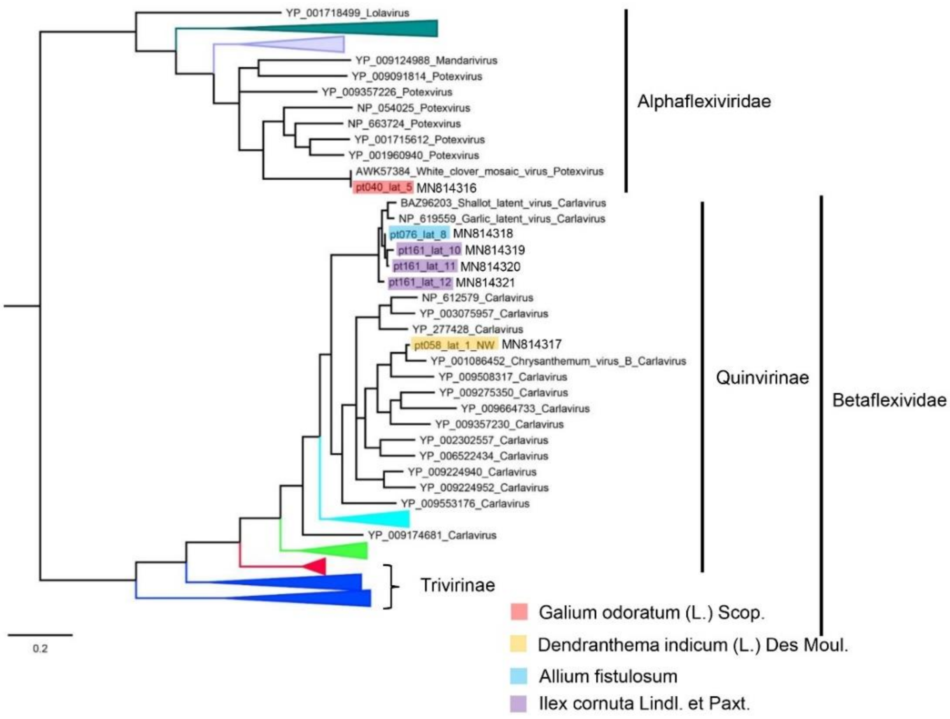

Supplementary Figure 26. The phylogenetic tree and viral genome organization of plant viruses belonging in the family *Tombusviridae*. Figure legend is the same as Supplementary Figure 3 and 4.

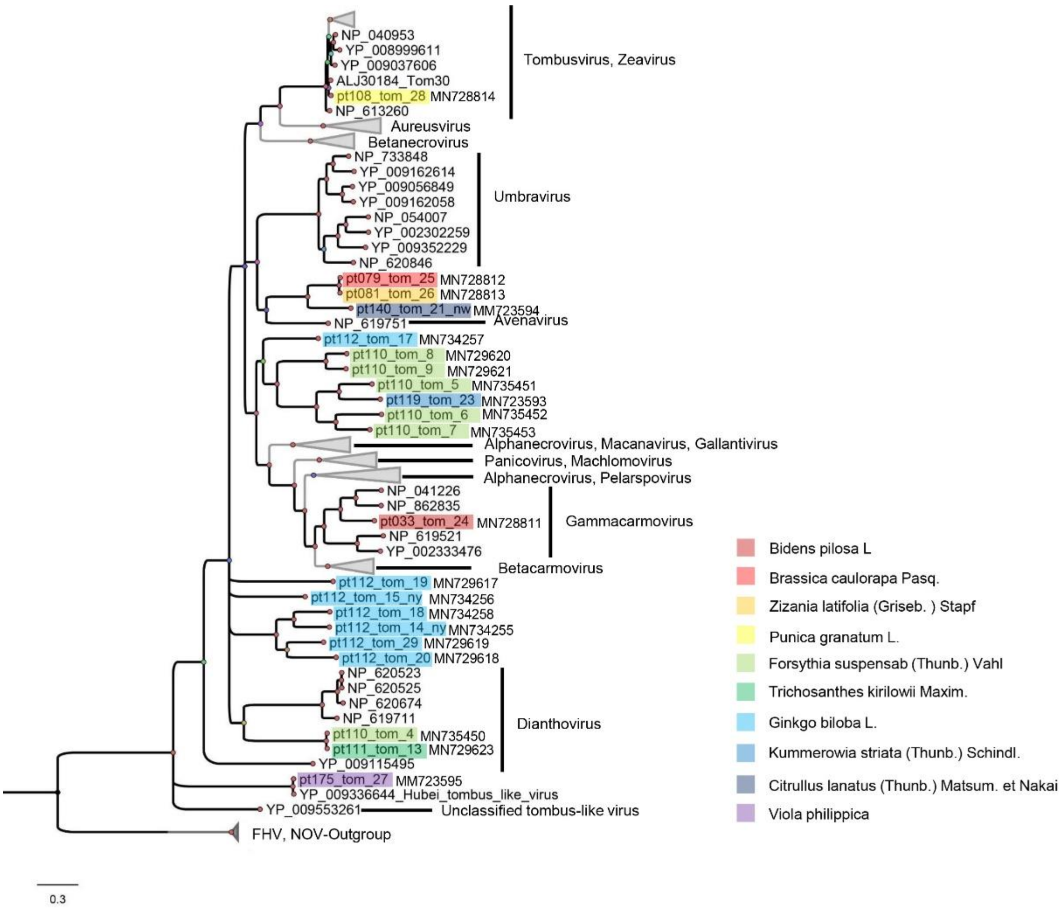

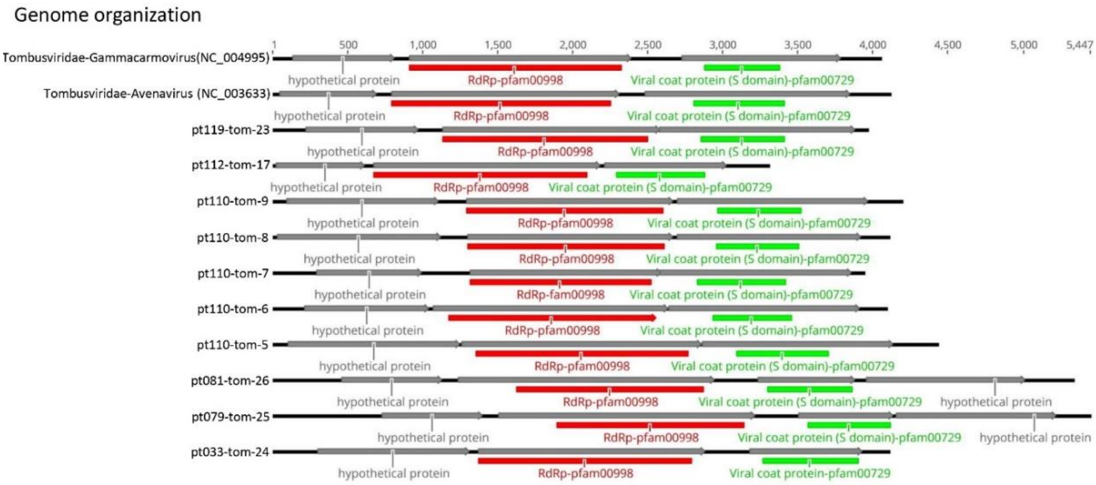

Supplementary Figure 27. The phylogenetic tree of plant-associated letuo-like virus and viruses belonging in the family *Luteoviridae*. Figure legend is the same as Supplementary Figure 3.

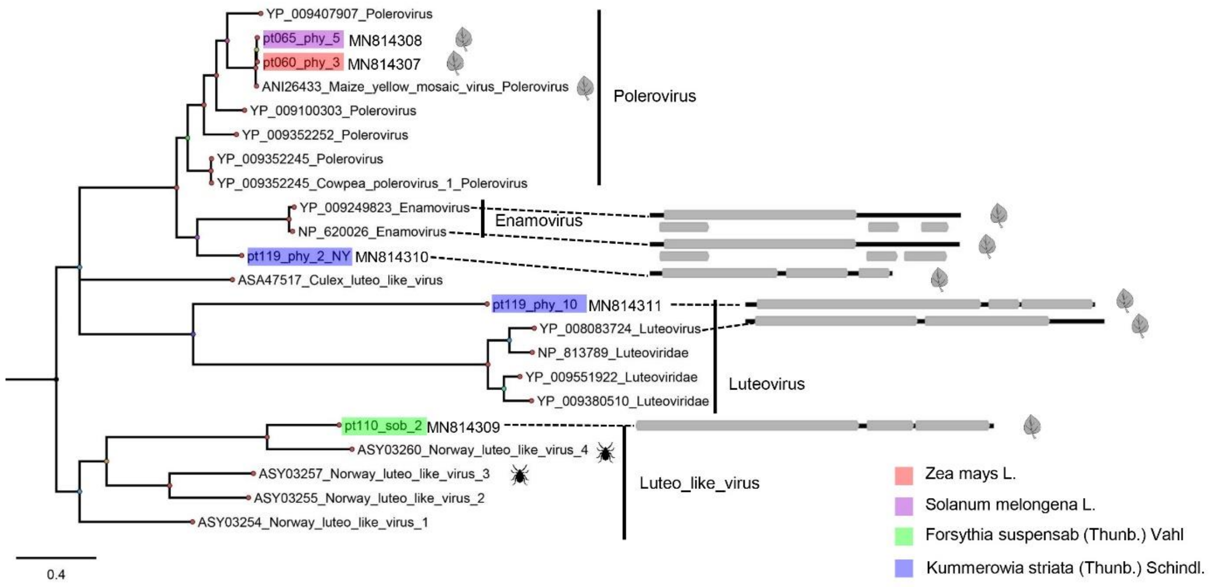

Supplementary Figure 28. The phylogenetic tree of plant-associated parti-like virus and viruses belonging in the family *Partitiviridae*. Figure legend is the same as Supplementary Figure 3.

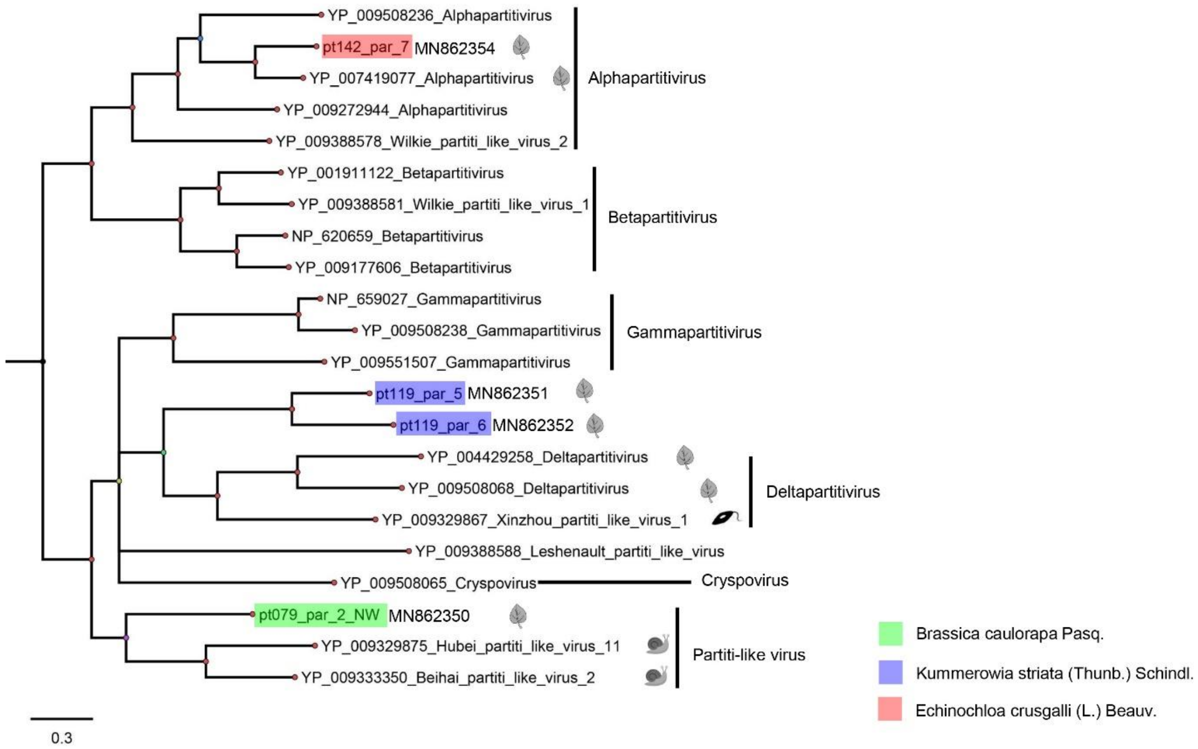

Supplementary Figure 29. The phylogenetic tree and viral genome organization of plant-associated Sobemo-like viruses. Figure legend is the same as Supplementary Figure 3 and 4.

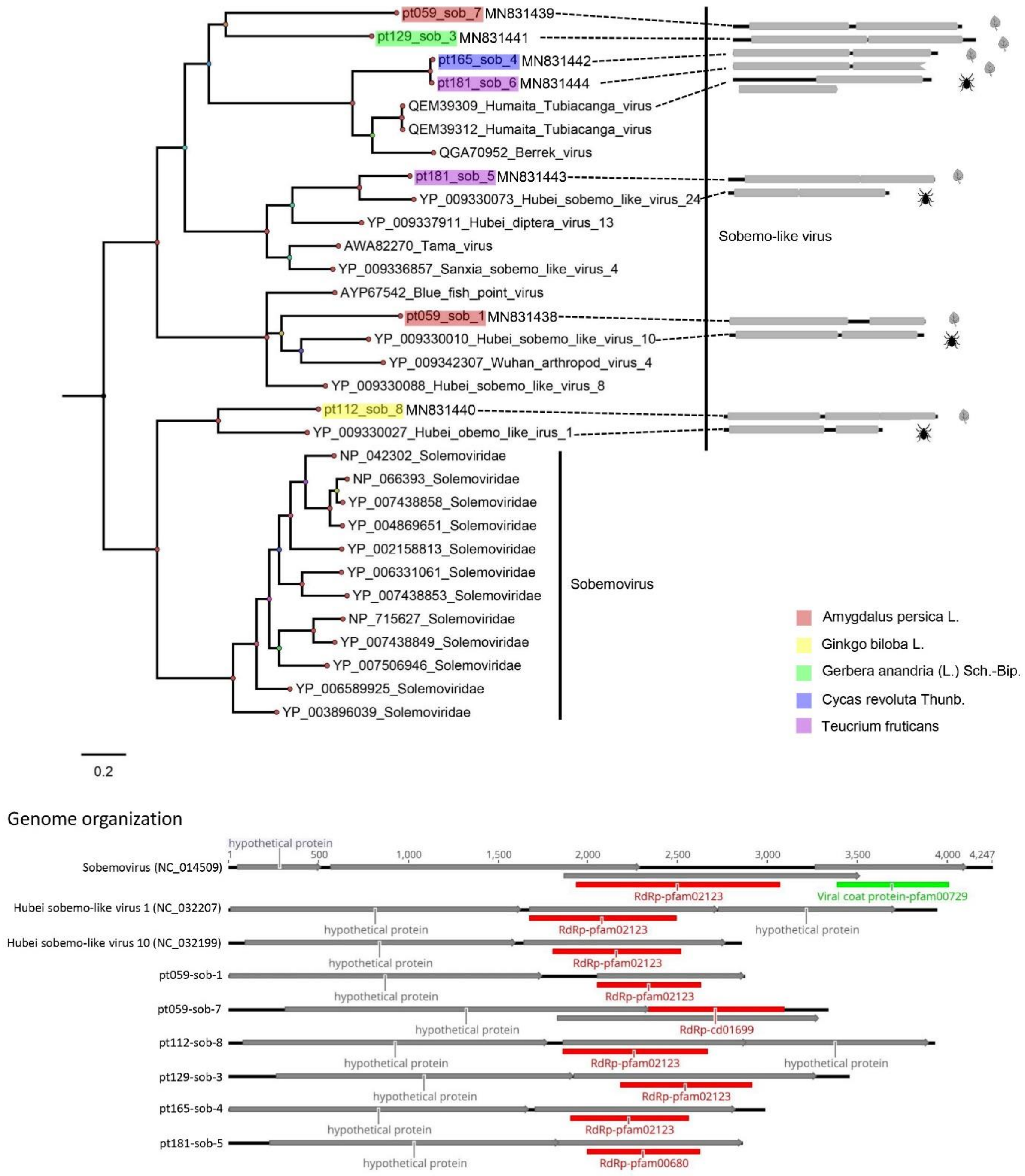

Supplementary Figure 30. The phylogenetic tree of plant-associated Rhabdo-like viruses. Figure legend is the same as Supplementary Figure 3.

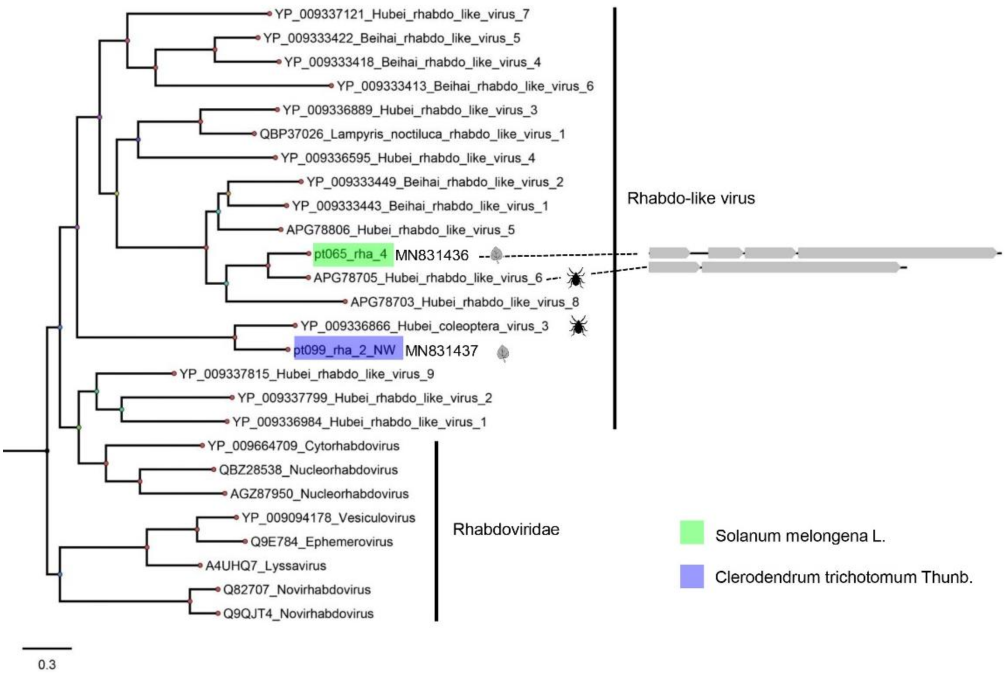

Supplementary Figure 31. The phylogenetic tree and viral genome organization of plant-associated Bufivirus-like viruses. Figure legend is the same as Supplementary Figure 3 and 4.

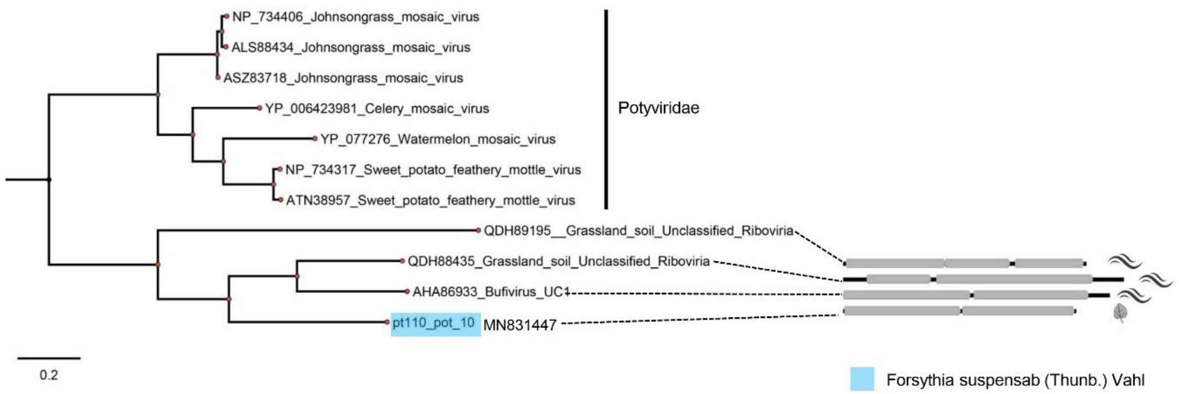

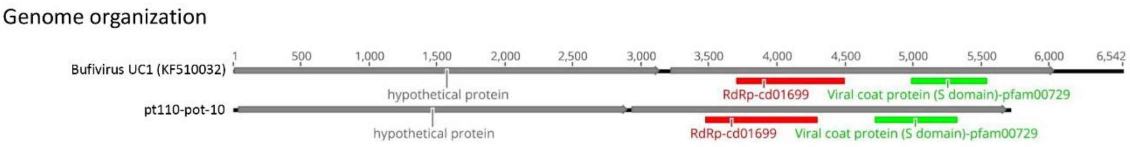

Supplement: Supplementary file 3 — Additional file 3. Fig. S2-31: The map for sampling sit of plant samples in this study and the detailed phylogenetic tree and genome structure of different viruses in this study. [file 40793_2022_453_MOESM3_ESM.pdf]
