# Supplementary material for: Expanding known viral diversity in plants: virome of 161 species alongside an ancient canal
Source: Environ Microbiome. 2022 Nov 27;17:58. doi: 10.1186/s40793-022-00453-x (PMC9703751; doi:10.1186/s40793-022-00453-x)
Supplement: Supplementary file 5 — Additional file 5. Fig. S32: The host assignment of plant-associated viruses by using BLASTx searching the contigs assembled from the NGS data of each plant species (those containing possible non-plant-infecting virus) against the total mitochondrial proteome database that were downloaded from GenBank. [file 40793_2022_453_MOESM5_ESM.pdf]

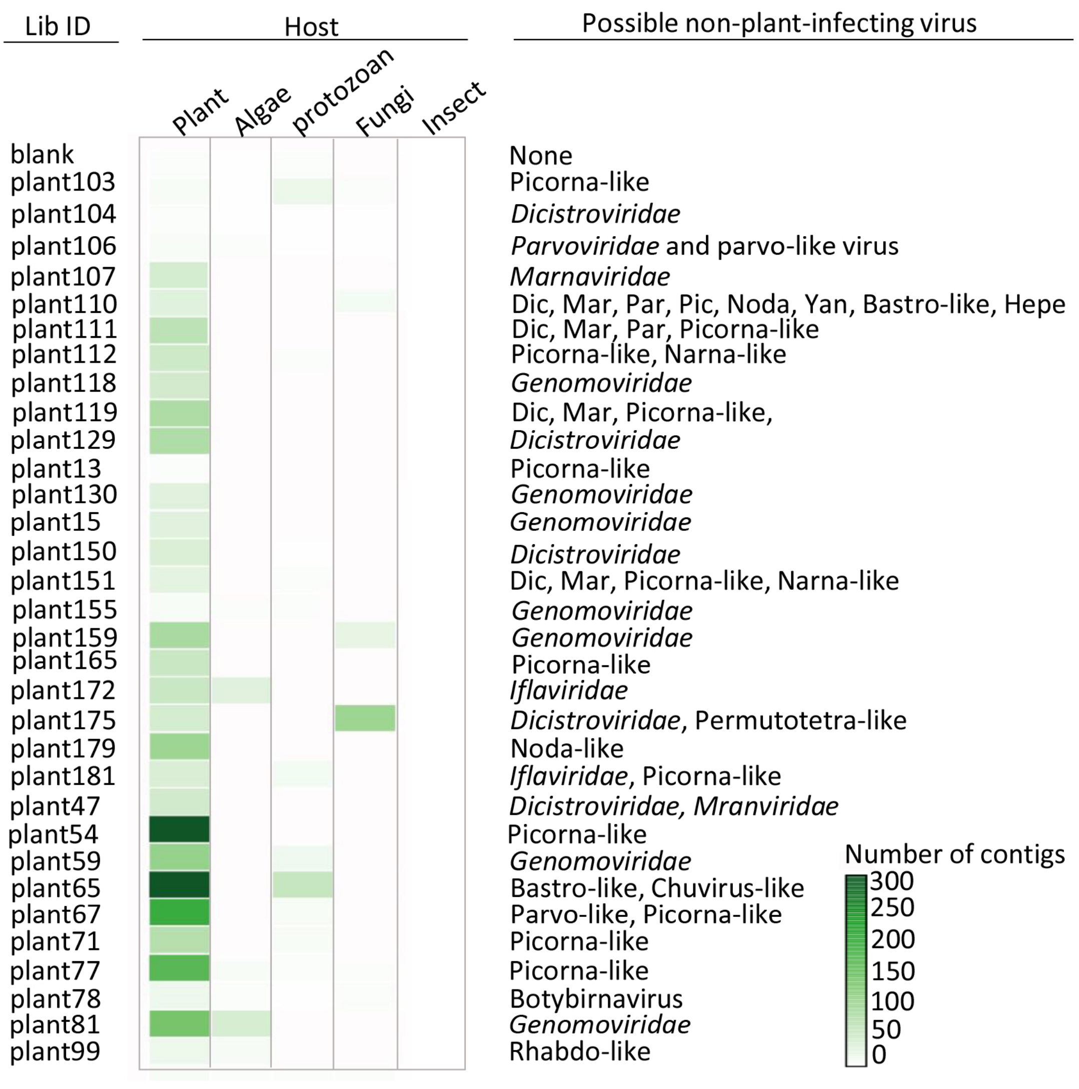

Dic: *Dicistroviridea*, Mar: *Marnaviridae*, Par: *Parvoviridae*, Pic: *Picornaviridae*, Noda: *Nodaviridae*, Yan: Yanvirus, Hepe: Hepe-like virus
